# Joint regression analysis of multiple traits based on genetic relationships

**DOI:** 10.1093/bioadv/vbad192

**Published:** 2024-01-04

**Authors:** Ann-Sophie Buchardt, Xiang Zhou, Claus Thorn Ekstrøm

**Affiliations:** Department of Public Health, University of Copenhagen, 1014 Copenhagen, Denmark; Department of Biostatistics, University of Michigan, Ann Arbor, MI 48109, United States; Department of Public Health, University of Copenhagen, 1014 Copenhagen, Denmark

## Abstract

**Motivation:**

Polygenic scores (PGSs) are widely available and employed in genomic data analyses for predicting and understanding genetic architectures. Existing approaches either require information on SNP level, do not infer clusters of traits sharing genetic characteristic, or do not have any immediate predictive properties.

**Results:**

Here, we present geneJAM, which is a novel clustering and estimation method using PGSs for inferring a genetic relationship among multiple, simultaneously measured and potentially correlated traits in a multivariate GWAS.

Using graphical lasso, we estimate a sparse covariance matrix of the PGSs and obtain clusters of traits sharing genetic characteristics. We use the clusters to specify the structure of the error covariance matrix of a generalized least squares (GLS) model and use the feasible GLS estimator for estimating a linear regression model with a certain unknown degree of correlation between the residuals.

The method suits many biology studies well with traits embedded in some genetic functioning groups and facilitates development of the PGS research. We compare the method with fully parametric techniques on simulated data and illustrate the utility of the methods by examining a heterogeneous stock mouse data set from the Wellcome Trust Centre for Human Genetics. We demonstrate that the method successfully identifies clusters of traits and increases precision, power, and computational efficiency.

**Availability and implementation:**

GeneJAM is implemented in R and available at: https://github.com/abuchardt/geneJAM.

## 1 Introduction

The motivation for this paper is the problem of jointly analysing multiple, simultaneously measured quantitative traits in a genome-wide association study (GWAS), i.e. a multivariate GWAS. Multivariate GWASs have gained attention in genetic studies as it offers several advantages over analysing each trait in a separate GWAS (Galesloot *et al.* 2014). While single-trait GWASs have found numerous genetic variants associated with complex diseases (MacArthur *et al.* 2016), traits are often correlated, e.g. due to pleiotropic genes, and therefore, a joint modelling approach can be used to increase precision and power (Schmitz *et al.* 1998). Furthermore, these variants typically have a small effect and correspond to a small fraction of truly associated variants; therefore, they have limited predictive power (Yang *et al.* 2010, Dudbridge 2013).

While a univariate GWAS cannot exploit potential correlation between traits, carrying out a multivariate genome-wide association (GWA) analysis may vastly increase the statistical and computational complexity of the analyses. For example, an unconstrained correlation matrix for *q* traits requires q(q − 1)/2 correlation parameters. Such large numbers of parameters require very large data sets and present considerable computational challenges.van Rheenen *et al.* (2019) provide a review of the definition, estimation, interpretation, and uses of genetic correlations, with a focus on applications to human disease. The specific question of shared genetics has been addressed many times. For example, Julienne *et al.* (2021) conduct multi-trait analyses of GWAS summary statistics from 36 human phenotypes combining association tests and clustering to detect the shared and specific genetic substructures underlying those phenotypes and to explore the links between those substructures and biological pathways and diseases. Pickrell *et al.* (2016) present detection and interpretation of shared genetic influences on 42 human traits, and Watanabe *et al.* (2019) address the question of the extent of pleiotropy across the (human) genome and variation in genetic architecture across traits.

Different multivariate GWA methods have been formulated for simultaneously testing marker association with multiple traits influenced by pleiotropic genes. Most of these methods require information on SNP level. For example, Kang *et al.* (2008) propose efficient mixed-model association (EMMA), which corrects for population structure and genetic relatedness. This method for exact computation of standard test statistics is computationally impractical for even moderate-sized GWASs. Zhou and Stephens (2012) present an efficient exact method, genome-wide efficient mixed-model association (GEMMA), which is claimed to be approximately *N* times faster with *N* being the sample size. Both methods are implemented in the R packages emma and gemma, respectively, but the implemented algorithms are not suitable for large-scale problems in terms of the number of traits (Zhou and Stephens 2014). Korte *et al.* (2012) also extend the linear mixed-model approach to carry out GWAS of correlated phenotypes, deriving a fully parameterized multi-trait mixed model (MTMM) that considers both the within-trait and between-trait variance components simultaneously for multiple traits. MTMM has been implemented in a set of R scripts (MTMM) for carrying out GWAS. These scripts rely on the software ASREML (Gilmour *et al.* 2015) for the estimation of the variance components. O’Reilly *et al.* (2012) propose the MultiPhen method which performs GWAS on multiple phenotypes by identifying the linear combination of the phenotypes most associated with genotype at each SNP. This is achieved by reversing the regression, such that genotype is regressed on phenotype, rather than phenotype-on-genotype as in the standard GWAS approach. Their test for association is a likelihood ratio test for model fit, which provides a p-value for evidence of association between the SNP and the phenotypes. The method is implemented in the R package MultiPhen by Coin *et al.* (2020). Wang *et al.* (2016) propose an “optimal” maximum heritability test (MHT-O) to test the association between multiple traits and a single variant. Both the MultiPhen and the MHT-O test association between multiple traits and a genetic variant, and they do not infer clusters of traits sharing genetic characteristic nor do they have any immediate predictive properties. The most used approach today might be cross-trait linkage disequilibrium score regression (LD Score) regression (Bulik-Sullivan *et al.* 2015a), which quantifies the contribution of each polygenic signal by examining the relationship between test statistics and linkage disequilibrium (LD). A software tool for LD Score estimation and estimation of variance components from summary statistics is implemented in python (Bulik-Sullivan *et al.* 2015b). However, the method provides genetic correlation estimates of pairwise combinations of traits only, and it provides no solution for further predictive modelling of the traits. Cichonska et al. (2016) present a computational framework called metaCCA for summary statistics-based analysis that allows multivariate representation of both genotype and phenotype. It extends the statistical technique of canonical correlation analysis (CCA) to the setting where original individual-level records are not available, and employs a covariance shrinkage algorithm to achieve robustness. The approach is implemented in the R package metaCCA. However, in the in the case of multi-SNP-multi-trait analysis where a set of genetic variants is tested for an association with a set of phenotypic variables a genotypic correlation structure containing correlations between SNPs is needed. Zhu et al. (2015) propose a general approach that can integrate association evidence from summary statistics of multiple traits, either correlated, independent, continuous, or binary traits, which might come from the same or different studies. They allow for trait heterogeneity effects. Population structure and cryptic relatedness can also be controlled. The association tests for all the SNPs are performed with the software Plink (Purcell *et al.* 2007), but to our knowledge, software for the proposed general statistics is not available. Shi *et al.* (2017) introduce *ρ*-HESS, a technique to quantify the correlation between pairs of traits due to genetic variation at a small region in the genome. Their approach requires GWAS summary data only and makes no distributional assumption on the causal variant effect sizes while accounting for linkage disequilibrium (LD) and overlapping GWAS samples. The authors provide stand-alone software.

In the case of correlated traits, the ordinary least squares (OLS) estimator is unbiased, but inefficient. Instead we may use the *feasible generalized least squares (FGLS)* estimator. The feasible estimator is, provided the covariance matrix is consistently estimated, asymptotically more efficient for large samples under heteroskedasticity. A further challenge is that the total number *p* of features (e.g. SNPs) is usually larger than the sample size *N* when working with genomic data. In this case, the design matrix does not have full rank and the OLS estimator cannot be computed. Various methodologies deal with this problem as well as how to generate the weights of the SNPs and how to determine which s≤p features should be included (e.g. Euesden *et al.* (2014); Wray *et al.* (2014)). To meet these challenges, we introduce *polygenic scores (PGSs)*. Our goal is to use PGSs to both perform dimensionality reduction and identify clusters of traits to approximate the *structure* of the error covariance matrix of a linear regression model. We can then use the FGLS estimator for estimating the unknown parameters. PGSs are linear transformations of multiple genetic variants to scores that *summarize* the estimated effect of, e.g. SNPs. Often they are calculated as a weighted sum of SNPs, i.e. they are constructed from the “weights” derived from a GWAS, or from some form of machine learning algorithm. This way, a PGS reflects an estimated genetic predisposition for a given trait without taking environmental factors into account and can be used as a numeric predictor for that trait. We note that the predictive power of PGSs increases with the sample size of the underlying GWAS.

The objective of this paper is to propose a computationally efficient method for inferring a genetic relationship among a large number of simultaneously measured and potentially correlated traits in a multivariate GWAS. The method simultaneously analyses multiple correlated quantitative traits in clusters that share some genetic component under the assumption that the trait values in a cluster follow a multivariate normal distribution.

We propose a versatile tool called a joint analysis of multiple traits based on genetic relationships (*geneJAM*), which implements the method of finding clusters of correlated outcome components (traits) that share some genetic component by means of PGSs. The PGS method (Dudbridge 2013) aggregates the effects of variants across the genome to estimate heritability, infer genetic overlap between traits, and predict phenotypes based on the genetic profile. The approach is motivated by utilizing the widely available PGSs (e.g. Lambert *et al.* 2021) as numeric predictors to identify conditional independences of traits giving rise to clusters of traits, and, thereby, being able to analyse the data combined in clusters and increase precision and power and gain computational advantages. By estimating a sparse version of the precision matrix of the PGSs, approximate zeroes induce conditional independence which enables us to infer clusters of traits sharing genetic characteristics. Having identified the clusters, we are able to jointly analyse the traits, thereby increasing precision and power. We propose a simple approach: having estimated the precision matrix of the PGSs, we are able to approximate clusters of traits that share some genetic component. Thus, we are able to specify the structure of the error covariance matrix of a linear regression model when there is a certain unknown degree of correlation between the residuals and use the feasible generalized least squares (FGLS) estimator for estimating the unknown parameters.

In section 2, we introduce the conceptual framework of the geneJAM method and the theoretical foundation including linear regression models, the concept of PGSs, conditional independence, FGLS, and sparsity of correlation matrices. We study our method and other techniques on real data, as well as simulated data, in section 3. Specifically, we analyse a heterogeneous stock mouse data set (Valdar *et al.* 2006), from the Wellcome Trust Centre for Human Genetics. The methods are implemented using the R packages MESS by Ekstrøm (2019) and glasso by Friedman *et al.* (2018); the R code is part of our package geneJAM which is available online, see (Buchardt 2022). We conclude with a discussion and recommendations in section 4.

## 2 Method

We focus on linear regression models, which are suitable when the outcome is quantitative, and ideally when the error distribution is Gaussian. We consider *N* independent samples, $y_{1},\ldots ,y_{N}\in R^{q}$, of a *q*-dimensional random variable distributed according to a multivariate normal distribution. We define a matrix, **Y**, from the *N* observations of the *q* outcome components, $Y={\left(y_{1},\ldots ,y_{N}\right)}^{⊤}\in R^{N\times q}$. We also define a matrix $X={\left(x_{1},\ldots ,x_{N}\right)}^{⊤}\in R^{N\times p}$ of *N* samples of *p* associated features, e.g. a mixture of environmental and genetic factors. We assume that the data-generating process can be described by a linear regression model of the form

(1) Y=M + XC + E,

where $M=1_{N}\mu^{⊤}\in R^{N\times q}$ is a matrix of intercepts with $\mu ={\left(\mu_{1},\ldots ,\mu_{q}\right)}^{⊤},\;C\in R^{p\times q}$ is a matrix of regression coefficients, and $E\in R^{N\times q}$ are independent Gaussian random errors.

However, we are interested in a more constrained scenario, where we consider information on *polygenic score (PGS)* level. PGSs are linear transformations between multiple genetic variants to scores that *summarize* the estimated effect of, e.g. SNPs. PGSs are widely used and available in many fields such as animal breeding and plant breeding, and they are also used in human genetics. Typically, they are calculated as a weighted sum of trait-associated alleles, which are one of two, or more, forms of a given gene variant. That is, they are constructed from the “weights” derived from a GWAS, or from some form of machine learning algorithm. In a GWAS, a set of genetic markers (usually SNPs) is genotyped on a training sample, and effect sizes are estimated for each marker’s association with the trait of interest. Thus, a PGS reflects an estimated genetic predisposition for a given trait without taking environmental factors into account, and it can be used as a numeric predictor for that trait. For the purpose of completeness, we present a simple method of construction here.

While geneJAM can leverage external PGSs derived from independent data to improve performance, it is also directly applicable to situations where no external PGSs are available.

### 2.1 Polygenic scores

In general, the weights used for the PGSs are estimated using some form of regression analysis, and since the total number of features *p* is usually larger than the sample size *N*, one cannot use OLS for estimating the parameters simultaneously. Various methodologies deal with this problem as well as how to generate the weights of the SNPs and how to determine which features should be included (e.g. Euesden *et al.* (2014); Wray *et al.* (2014)). To keep the setup simple, we define, for each trait l=1,…,q, weights $w_{1l},\ldots ,\;w_{pl}$ as the marginal effects on the trait $y_{l}$, estimated separately from a univariate simple linear regression model. This way, we have to do p·q separate univariate simple linear regression analyses, but we avoid making dimensionality reduction of the SNPs before computing the PGSs. That is, for each outcome component $y_{l}\in R^{N},\;l=1,\ldots ,q$, and each feature $x_{j}\in R^{N},\;j=1,\ldots ,p$, we estimate a univariate simple linear regression model of the form

(2) $$y_{l}=w_{0l}\;+\;x_{j}w_{jl}\;+\;e_{l},$$

where $w_{0l}\in R$ is the intercept, $w_{jl}\in R$ is a regression coefficient, and $e_{l}\in R^{N}$ is a vector of independent Gaussian random errors. For ease of notation, we label all regression coefficients as elements in a *p *×* q* matrix **W**, which is not to be mistaken for a matrix of coefficients from a multivariate multiple linear regression. Finally, we define the PGS for each outcome component l=1,…,q and each individual i=1,…,N by

$$z_{il}=\sum_{j=1}^{p}x_{ij}{\hat{w}}_{jl},$$

where ${\hat{w}}_{jl}$ is the maximum likelihood estimate of *w_jl_*.

In the following, we assume that a set of PGSs has been provided—one for each outcome—and we use this set of estimated PGSs as numeric predictors (features). That is, we assume that we have access to *N* samples of exactly one feature per trait, $z_{1},\ldots ,z_{N}\in R^{q}$.

We let $\vec{Y}=\text{vec}(Y^{⊤})\in R^{Nq}$ denote the row-wise vectorization of **Y** obtained by stacking the columns of the matrix $Y^{⊤}$, i.e. $\vec{Y}={\left(y_{11},y_{12},\ldots ,y_{1q},y_{21},y_{22},\ldots ,y_{2q},\ldots ,y_{N1},y_{N2},\ldots ,y_{Nq}\right)}^{⊤}$. Now, we consider a linear regression model on the form

(3) $$\vec{Y}=\vec{Z}\vec{B}\;+\;\vec{E},$$

where $\vec{B}\in R^{2q}$ denotes the row-wise vectorization of a matrix $B\in R^{2\times q}$ of unknown intercepts and regression coefficients such that $\vec{B}={\left(\xi_{1},\beta_{1},\xi_{2},\beta_{2},\ldots ,\xi_{q},\beta_{q}\right)}^{⊤}$. $\vec{Z}\in R^{Nq\times 2q}$ is a corresponding design matrix of features, and $\vec{E}\in R^{Nq}$ is a vector of Gaussian random errors. To allow for among-trait correlation, we assume that

$$\vec{E}∼N_{Nq}\left(0_{Nq},\Omega \right),$$

where $0_{Nq}\in R^{Nq}$ is a vector of zeros and $\Omega \in R^{Nq\times Nq}$ is a general covariance matrix. Possible structures for Ω may be a block diagonal matrix, a diagonal matrix, or, more importantly, a general positive definite matrix. This flexibility is essential to our method, since the outcome that our method is targeted towards, is supposed to be both correlated and heteroscedastic across outcome components. In particular, we assume that the covariance matrix, Ω, is a block diagonal matrix on the form

(4) $$\Omega =\left[\begin{array}{cccc} R & 0 & \cdots & 0\\ 0 & R & \cdots & 0\\ \vdots & \vdots & \ddots & \vdots \\ 0 & 0 & \cdots & R \end{array}\right],$$

where $R\in R^{q\times q}$ are the residual covariances among traits within each individual. If the covariance matrix is known, the generalized least squares (GLS) estimator is the best linear unbiased estimator of the unknown parameters $\vec{B}$ in a linear regression model when there is a certain known degree of correlation between the residuals. In these cases, ordinary least squares (OLS) and weighted least squares (WLS) can be statistically inefficient, or even give misleading inferences (Baltagi 2008). In our case, the among-trait and within-individual correlation, Ω, of the errors is unknown, but we can get a consistent (in terms of structure) estimate using the feasible generalized least squares (FGLS) estimator. In general terms, the FGLS procedure consists of two steps: first, a linear regression model is estimated by OLS, and the residuals are used to build an estimator of the covariance matrix of the errors. Second, using the estimator of the covariance matrix of the errors, the unknown regression coefficients are estimated using WLS, which generalizes OLS and allows the covariance matrix of the errors to be different from an identity matrix. If the estimator of the covariance matrix of the errors is a consistent estimator, like the OLS, then the FGLS estimator of the unknown regression coefficients is a consistent estimator (Baltagi 2008). The FGLS estimator is discussed in more detail in subsection 2.2.

Our initial motivation for considering PGSs is the presumption that some of the variability observed in a trait is attributable to genetic differences. Therefore, the traits may be correlated via the PGS, and it may be reasonable to assume that the underlying independence relations for the traits can be approximated by the relations observed in the PGSs.

### 2.2 The geneJAM method

We provide a method which identifies blocks in the correlation matrix of PGSs via the graphical lasso with regularization parameter *τ* and models the relationship between traits and associated PGSs by linear regression. To obtain a better fit, the FGLS estimator is used to provide a consistent estimate of the covariance, **R**, of the errors with a block structure identified at a given *τ*, and, then, weighted least squares (WLS) is used for estimating the unknown parameters in a linear regression model. This way, we identify clusters of potentially correlated outcome components by means of PGSs, under the assumptions that features which are marginally highly associated with a trait should carry more weight in a joint analysis of traits and that much of the variability observed in a trait is attributable to genetic differences, i.e. heritability, such that the traits may be correlated via the PGS.

We assume that we have *N* observations of the multivariate outcome $Y\in R^{N\times q}$ with associated PGSs, $Z=\left(z_{1},\ldots ,z_{q}\right)\in R^{N\times q}$, for each trait individually, and that the PGSs are from the same population as the data at hand but estimated from a different sample. If existing PGSs for a trait have not been provided, we refer to subsubsection 3.1.1 for a brief introduction to generating PGSs and to Dudbridge (2013) for a comprehensive survey and a pointer to computing PGSs. We assume that the collection of PGSs can be approximated by a multivariate Gaussian distribution with mean μ and covariance Σ.

If we represent the system of PGSs as an undirected graph G, where the PGS for a certain trait is represented by a node, then an edge represents related pairs of traits in terms of PGSs. Moreover, a *connected component* of a graph, which is a maximal sub-graph in which there exists a path between any two nodes, represents a cluster of related traits. In a probabilistic graphical model, the conditional independence structure is encoded in a graph, since the presence/absence of edges encodes conditional independence relations among associated nodes (Lauritzen 1996).

Identifying connected components of the graph G is one of the core functionalities of the geneJAM method: A useful property of the multivariate Gaussian distribution is that, for a matrix of random variables following a multivariate Gaussian distribution, the *ij*th component of the corresponding precision matrix (the matrix inverse of the covariance matrix) is zero if and only if the variables *i* and *j* are conditionally independent, given the others. This is an implication of the basic property of the multivariate Gaussian distribution being closed under conditioning (Lauritzen 1996). We wish to exploit this property: under the assumption that much of the variability observed in a trait is attributable to genetic differences, i.e. heritability, the traits may be correlated via the PGSs. That is, by assuming that the PGSs follow a multivariate Gaussian distribution, approximating the precision matrix, **P**, of the PGSs and further sparsity, we are able to identify blocks of zeros in the precision matrix, and, thus, potential connected components, which correspond to clusters of traits sharing some genetic characteristics.

As a way of estimating a sparse precision matrix, we consider the *graphical lasso* (Friedman *et al.* 2007). The graphical lasso estimates a sparse precision matrix using a lasso penalty, and the regularization path is computed at a grid of values for the regularization parameter *ρ*. Thus, from the sparse precision matrix, ${\hat{P}}_{\left(\rho \right)}$, of the PGSs estimated by graphical lasso at a given value of *ρ*, we obtain a clustering, i.e. a set of $C_{\left(\rho \right)}$ connected components. We assume that the residual covariances among traits within each individual, **R**, exhibit this clustering as well.

Let us elaborate on how the graphical lasso is used to construct an estimator of the correlation matrix. For computational reasons, the PGSs should be centred before the graphical lasso optimization problem is solved, such that each column has mean zero. That is, $\frac{1}{N}\sum_{i=1}^{N}z_{il}=0,\;l=1,\ldots ,q$. We denote by Σ the *q *×* q* positive-definite covariance matrix of the matrix, **Z**, of scaled and centred PGSs. From this, we estimate a sparse precision matrix using a lasso ($\ell_{1}$) penalty via the graphical lasso. For a precision matrix **P** and an empirical correlation matrix **S,** the graphical lasso maximizes the penalized log-likelihood

$$\text{log}\;\left(\det\left(P\right)\right)\;-\;\text{tr}\left(SP\right)\;-\;\rho ||P|{|}_{1}$$

over non-negative definite matrices **P**. Here det denotes the determinant, tr denotes the trace, and $||P|{|}_{1}=\left(\sum_{i=1}^{q}|p_{i}|\right)$ is the $\ell_{1}$ norm. In order to show how to infer connected components, we recall the definition of an adjacency matrix. By Peters *et al.* (2018) Definition B.3, we can represent a directed graph G=(V,E) over *q* nodes with a binary *q *×* q* matrix **A** (taking values 0 or 1):

$$a_{ij}=1\Leftrightarrow (i,j)\in E.$$

**A** is called the *adjacency matrix* of G. If the graph is undirected, i.e. all of its edges are bidirectional, the adjacency matrix is symmetric. In words, the adjacency matrix of an undirected graph is a symmetric square matrix with zeros on the diagonal, and the non-zero elements (usually ones) of the matrix represent pairs of nodes which are adjacent. We are able to generate an adjacency matrix from the estimated sparse precision matrix, ${\hat{P}}_{ij}$ by letting *A_ij_* = 1 if ${\hat{P}}_{ij}\neq 0$ for i≠j and zero otherwise. We note that a sufficiently small penalization, τ→0, most likely results in a dense version of the precision matrix, corresponding to a fully connected graph with no conditionally independent random variables. Similarly, a sufficiently large penalization, τ→∞, most likely results in a sparse version of the precision matrix, corresponding to a graph with no edges and only conditionally independent random variables. What is considered a good choice of regularization parameter, however, depends on whether the goal is prediction accuracy, increasing precision of estimates, gain in power, or recovering the right model for interpretation purposes. In conclusion, by using the graphical lasso we are able to obtain an approximation of the precision matrix, ${\hat{P}}_{\left(\tau \right)}$, of the PGSs at a grid of values for the regularization parameter *τ*. From these, we construct corresponding adjacency matrices, ${\hat{A}}_{\left(\tau \right)}$, each of which represents a set of connected components $C_{\left(\tau \right)}$.

For the first step in the FGLS estimation of **R,** we define sub-vectors $b_{l}={\left(\xi_{l},\beta_{l}\right)}^{⊤}\in R^{2},\;l=1,\ldots ,q$, of $\vec{B}$ and design matrices $Z_{l}\in R^{N\times 2}$, where a constant term is included, such that the first column of $Z_{l}$ is a column of ones allowing estimation of the intercept, while the following column contains the feature (PGS) associated with the corresponding trait value. An OLS estimator of $b_{l}$ is calculated from a linear regression model for each trait, l=1,…,q, separately:

$$y_{l}=Z_{l}b_{l}\;+\;e_{l},$$

where $e_{l}\in R^{N},\;l=1,\ldots ,q$, are independent Gaussian random errors $e_{l}∼N_{N}(0_{N},\sigma_{l}^{2}I_{N\times N}),$ with $\sigma_{l}^{2}>0$. The OLS estimator of $b_{l},\;l=1,\ldots ,q$, is

$${\hat{b}}_{l}^{O}={\left(Z_{l}^{⊤}Z_{l}\right)}^{-1}Z_{l}^{⊤}y_{l}.$$

From these estimates, the corresponding estimated residuals

$${\hat{u}}_{l}={\left(Y\;-\;Z{\hat{B}}^{O}\right)}_{l},$$

for all l=1,…,q, are obtained. Here, $Z\in R^{N\times (q+1)}$ is a corresponding design matrix of features, where a column of ones is included to allow for estimation of the intercept. Now, for each value of the regularization parameter *τ*, we construct an estimate, ${\hat{R}}_{\left(\tau \right)}^{O}$, of the covariance of the errors by computing the covariance of the OLS estimated residuals, $\hat{U}=\left({\hat{u}}_{1},\ldots ,{\hat{u}}_{q}\right)\in R^{N\times q}$, in the same connected component. That is,

$${\hat{R}}_{g(\tau )}^{O}=\text{cov}\left({\hat{U}}_{g(\tau )}\right),$$

where ${\hat{R}}_{g(\tau )}^{O}$ and ${\hat{U}}_{g(\tau )}$ are the sub-matrices and of ${\hat{R}}_{\left(\tau \right)}^{O}$ and $\hat{U}$, respectively, corresponding to the connected components $g(\tau )=1,\ldots ,C_{\left(\tau \right)}$ at a given value of *τ*. From ${\hat{R}}_{\left(\tau \right)}^{O}$, we construct an estimate, ${\hat{\Omega }}_{\left(\tau \right)}^{O}$, of Ω on the form (4). Step one of the FGLS procedure is then fulfilled.

In the second step in the FGLS estimation, we build the FGLS estimator, ${\hat{\vec{B}}}_{\left(\tau \right)}^{F}\in R^{2q}$, using WLS:

$${\hat{\vec{B}}}_{\left(\tau \right)}^{F}={\left({\vec{Z}}^{⊤}{\left({\hat{\Omega }}_{\left(\tau \right)}^{O}\right)}^{-1}\vec{Z}\right)}^{-1}{\vec{Z}}^{⊤}{\left({\hat{\Omega }}_{\left(\tau \right)}^{O}\right)}^{-1}\vec{Y}.$$

The procedure is iterated with the first iteration given by

$$\begin{array}{c} {\hat{U}}^{F(1)}=\left(Y-{\hat{Y}}_{\left(\tau \right)}\right)\\ {\hat{R}}_{g(\tau )}^{F(1)}=\text{cov}\left({\hat{U}}_{g(\tau )}^{F(1)}\right)\\ {\hat{\Omega }}_{\left(\tau \right)}^{F(1)}=\text{diag}\left({\hat{R}}_{1}^{F(1)},\ldots ,{\hat{R}}_{C(\tau )}^{F(1)}\right)\\ {\hat{\vec{B}}}_{\left(\tau \right)}^{F(2)}={\left({\vec{Z}}^{⊤}{\left({\hat{\Omega }}_{\left(\tau \right)}^{F(1)}\right)}^{-1}\vec{Z}\right)}^{-1}{\vec{Z}}^{⊤}{\left({\hat{\Omega }}_{\left(\tau \right)}^{F(1)}\right)}^{-1}\vec{Y} \end{array}$$

where ${\hat{Y}}_{\left(\tau \right)}\in R^{N\times q}$ is a by-row matrix transformation of

$${\hat{\vec{Y}}}_{\left(\tau \right)}=\vec{Z}{\hat{\vec{B}}}_{\left(\tau \right)}^{F}.$$

This estimation of Ω is iterated to convergence, and we obtain estimates ${\hat{\Omega }}_{\left(\tau \right)}$ and ${\hat{\vec{B}}}_{\left(\tau \right)}$. The standard error, ${\text{SE}}_{\left(\tau \right)}$, of the estimated coefficients is given by

$${\text{SE}}_{\left(\tau \right)}=\sqrt{\text{diag}\left({\left({\vec{Z}}^{⊤}{\left({\hat{\Omega }}_{\left(\tau \right)}\right)}^{-1}\vec{Z}\right)}^{-1}\right)}.$$

As we discuss below, choosing the value of the regularization parameter *τ* is an important issue in practice as it controls the amount of regularization of the precision matrix, i.e. the conditional independence structure of the PGSs.

An algorithmic overview of the method is shown in Algorithm 1.

### 2.3 Optimizing precision

Tuning of the regularization parameter *τ* is crucial since a large value of *τ* for the graphical lasso will make all variables conditionally independent, while a small value of *τ* will keep most variables in one cluster. We choose the regularization parameter for the purposes of increasing the precision of the estimates and note that it may be preferable to err on the side of complexity and choose less sparsity to obtain larger clusters, and thus more complex models, in order to further increase power and not fail to detect clusters in the data.

Since we aim at optimizing the precision, we use the standard error of the estimated coefficients to tune the geneJAM method. More specifically, for a given value of the regularization parameter, we compute the mean of the standard errors of estimates for traits which have been clustered. Traits which, at a certain level of regularization, are left as singletons stay as singletons as the regularization increases, and, since there is no regularization on singletons, they are modelled using a simple linear regression estimated by OLS. Hence, traits belonging to clusters of size one are irrelevant for the tuning. Therefore, for a given value, *τ*, of the regularization parameter, we compute the standard error ${\text{SE}}_{\left(\tau \right)}\in R^{q}$ and, for all traits which are in clusters of size strictly larger than one, we compute the average standard error (SE), i.e.

$${\bar{\text{SE}}}_{\left(\tau \right)}=\frac{1}{\left|G_{\left(\tau \right)}\right|}\sum_{l\in G_{\left(\tau \right)}}{\text{SE}}_{(\tau )l},$$

where $G_{\left(\tau \right)}$ is the index set of traits in connected components of size strictly larger than one and $\left|G_{\left(\tau \right)}\right|$ is the cardinality of $G_{\left(\tau \right)}$. We use the smallest value of ${\bar{\text{SE}}}_{\left(\tau \right)}$, over all tried values of *τ*, as our selection criterion for the regularization.

In practice, the regularization path is computed at a linear grid of values (possibly on the log scale) for the regularization parameter *τ*. A path is calculated ranging from the minimum absolute value of the covariance matrix of the PGSs, Σ, to the maximum column sum of the same covariance matrix. Note that this way, the regularization path is unique to the specific covariance matrix of interest. Although the geneJAM fits the model for all the values of *τ* by default, it stops early if, for the current *τ*, the sparse precision matrix estimated by the graphical lasso is a zero matrix (typically near the end of the path).

## 3 Results

We illustrate the utility of the geneJAM method presented in subsection 2.2. on both simulated and real data. The motivation for both sets of examples is to understand the performance of the method on the important challenges arising from simultaneously measured and potentially correlated traits. First, we use simulations to assess the ability to cluster traits under different levels of heritability and phenotypic correlation. Next, we assess the modelling performance of the method in terms of precision, computing time, and memory usage.

Most complex traits are highly polygenic; they are influenced by a large number of genetic variants with moderate effects, rather than a handful of variants with large effects (Price *et al.* 2015). This motivates the infinitesimal model (Fisher 1918) which assumes that a complex trait is influenced by an infinite number of loci, all of which have an infinitesimal effect. In practice, this typically results in the assumption of a Gaussian distribution of the additive genetic effects. The variance of these effects is the additive genetic variance *V_A_*, and a common way to evaluate how a population can respond to selection is through the ratio between the additive genetic variance and the total phenotypic variance (*V_P_*), namely the heritability *h*^2^, i.e.

$$h^{2}=\frac{V_{A}}{V_{P}}.$$

To control *V_A_* in a simulation study, the statistical assumptions of the infinitesimal model can be implemented using a linear mixed model (LMM), in which a random effect is included to reflect the variance of additive genetic origin. In its most simple form, this model can be written as (1), i.e.

Y=M + XC + E,

where, now, XC are random effects of additive genetic origin and **E** is a Gaussian noise matrix representing the effect due to environment and error. The assumption of the infinitesimal model implies that the additive genetic effects **C** can be represented as zero-mean multivariate Gaussian distributed with covariance matrix **H** of heritability and relatedness between traits, i.e. $C∼N\left(0,HV_{A}\right)$. Since the errors **E** follow a Gaussian distribution as well, it follows that the traits are also Gaussian. Furthermore, we may derive

$$V_{A}=\frac{h^{2}}{V(E_{l})-h^{2}},$$

for any l=1,…,q.

In the following simulation study, the noise is simulated from the standard Gaussian distribution, and the SNPs **X** are simulated from the binomial distribution with the number of trials, *n*, equal to two, and success probability *π*. This way, in the simple case of a single locus with two alleles denoted *A* and *a* with allele frequencies f(A)=π and f(a)=1 − π, respectively, the expected genotype frequencies under random mating are $f(AA)=\pi^{2}$ for the *AA* homozygotes, $f(aa)={\left(1\;-\;\pi \right)}^{2}$ for the *aa* homozygotes, and f(Aa)=2π(1 − π) for the heterozygotes. These frequencies correspond to the Hardy-Weinberg equilibrium with a minor allele frequency drawn from the continuous uniform distribution on the interval [0.1,0.5].

In order for us to assess the clustering ability of the method, we use the Rand index (RI) (Rand 1971) which measures the similarity between two clusterings. The Rand index represents the frequency of occurrence of agreements over the total pairs of traits, or the probability that two clusterings to be compared will agree on a randomly chosen pair. We apply the Rand index to the adjacency matrix **A**, which is a symmetric square matrix with zeros on the diagonal representing a finite undirected graph. The elements of the matrix represent pairs of edges which are adjacent (represented by a “1”) or not (represented by a “0”) in the graph. We generate the adjacency matrix from the estimated sparse precision matrix, ${\hat{P}}_{ij}$ by letting *A_ij_* = 1 if ${\hat{P}}_{ij}>0,\;i\neq j$, and zero otherwise.

We also assess the modelling performance of the method in terms of precision and computing time. Here, the goal is to show that by using the geneJAM to cluster and estimate we improve the precision of the estimates compared to using simple linear regressions or mixed models, and, given a clustering of the traits, estimation using FGLS is faster than the mixed-model estimation implemented in R in the lme4 package. Finally, we compute and compare the root mean squared error (RMSE) as a general purpose error metric for the numerical predictions:

$${\text{RMSE}}_{l}=\sqrt{\frac{1}{N}\sum_{i=1}^{N}{\left(y_{il}\;-\;{\hat{y}}_{il}\right)}^{2}},\;l=1,\ldots ,q.$$

We assess the clustering and precision of our method in a heterogeneous stock mouse data set (Valdar *et al.* 2006), from the Wellcome Trust Case Control Consortium (WTCCC).

### 3.1 Simulated data

Since the efficacy of a procedure depends on the true model generating the data, we simulate different combinations of heritability and phenotypic correlation. Each combination is tried as the ground truth, and we apply our method to each scenario separately.

#### 3.1.1 Sampling procedure

The sampling is a two-step procedure which goes as follows: first, we generate a sample with *N *=* *1000 observations of *p *=* *10 000 features, $X^{\left(0\right)}=\left(x_{1}^{\left(0\right)},\ldots ,x_{p}^{\left(0\right)}\right)\in {\left\{0,1,2\right\}}^{N\times p}$, in the Hardy–Weinberg equilibrium with a minor allele frequency of 0.3, as described above. From the features, we generate observations, $Y^{\left(0\right)}=\left(y_{1}^{\left(0\right)},\ldots ,y_{q}^{\left(0\right)}\right)\in R^{N\times q}$, by $Y^{\left(0\right)}=X^{\left(0\right)}C\;+\;E^{\left(0\right)}$, where $C\in R^{p\times q}$ is a matrix of multivariate Gaussian distributed genetic effects and $E^{\left(0\right)}=\left(e_{1}^{\left(0\right)},\ldots ,e_{q}^{\left(0\right)}\right)\in R^{N\times q}$ is a Gaussian noise matrix representing the effect due to environment and error. We are interested in examining the method at different levels of heritability $h_{l}^{2}$ and phenotypic correlation $\rho_{ll\prime },\;l,l\prime =1,\ldots ,q$, and we are able to control $h_{l}^{2}$ and $\rho_{ll\prime }$ by letting

(5) $$V(C_{l})=\frac{V_{A}}{V(X)}=\frac{h^{2}}{V(X)(V(E_{l})-h^{2})}$$

and

(6) $$\text{cov}\left(C_{l},C_{l\prime }\right)=\rho_{ll\prime }\frac{V(X)V(C_{l})\;+\;V(E_{l})}{E(X^{2})}.$$

From this initial sample, $\left(Y^{\left(0\right)},X^{\left(0\right)}\right)$, the PGSs, **Z**, are generated as follows: we estimate a univariate simple linear regression model of the form

$$y_{l}^{\left(0\right)}=x_{j}^{\left(0\right)}w_{jl}+e_{l}^{\left(0\right)},$$

where the scalars $w_{jl}\in R,j=1,\ldots ,p,\;l=1,\ldots ,q$, are regression coefficients and $e_{l}^{\left(0\right)}\in R^{N},l=1,\ldots ,q$ are vectors of independent Gaussian random errors. For ease of notation and implementation, the estimated regression coefficients, ${\hat{w}}_{jl},j=1,\ldots ,p,l=1,\ldots ,q$, are stored in a *p *×* q* matrix $\hat{W}$, which is not to be mistaken for a matrix of coefficients from a multivariate multiple linear regression. Next, we simulate a new sample of features, $X\in {\left\{0,1,2\right\}}^{N\times p}$, in the Hardy–Weinberg equilibrium with a minor allele frequency of 0.3, and from these features we generate new PGSs by $Z=X\hat{W}$. Finally, we simulate new observations by Y=XC+E, where $E\in R^{N\times q}$ is a Gaussian noise matrix. This way, we obtain traits and PGSs as if the PGSs were provided from an unrelated GWAS. We run the geneJAM procedure using the geneJAM function available online, see (Buchardt 2022). We repeat the data-generating process as well as the running of geneJAM 100 times. Thus, in total the method is evaluated on about 6000 simulations.

#### 3.1.2 Precision and clustering assessment

To assess the clustering ability of geneJAM, we apply the method when the data-generating process honours different levels of heritability and phenotypic correlation both ranging from 0 to 0.9. In addition, we compare the performance in terms of estimation of genetic correlation of the geneJAM and cross-trait LD Score regression.

First, we consider the scenario with no heritability of traits and no phenotypic correlation, i.e. $\rho_{ll\prime }=0$ and $h_{l}^{2}=0$ for all l,l′=1,…,10. We then consider different combinations of non-zero heritability of two traits and non-zero phenotypic correlation between these two traits. That is, we assume that $h_{1}^{2}=h_{2}^{2}>0$ and $\rho_{12}>0$ such that $\text{cov}(C_{1},C_{2})\neq 0$ and $\text{cov}(C_{l},C_{l\prime })\neq 0$ for $l,l\prime \neq 1,2,\;h_{1}^{2}=h_{2}^{2}\neq 0$, and $h_{l}^{2}=0$ for all l,l′≠1,2. Please note that, since the correlation matrix must be positive semidefinite, not all combinations of values of heritability and phenotypic correlation are viable.

**Figure 1.**
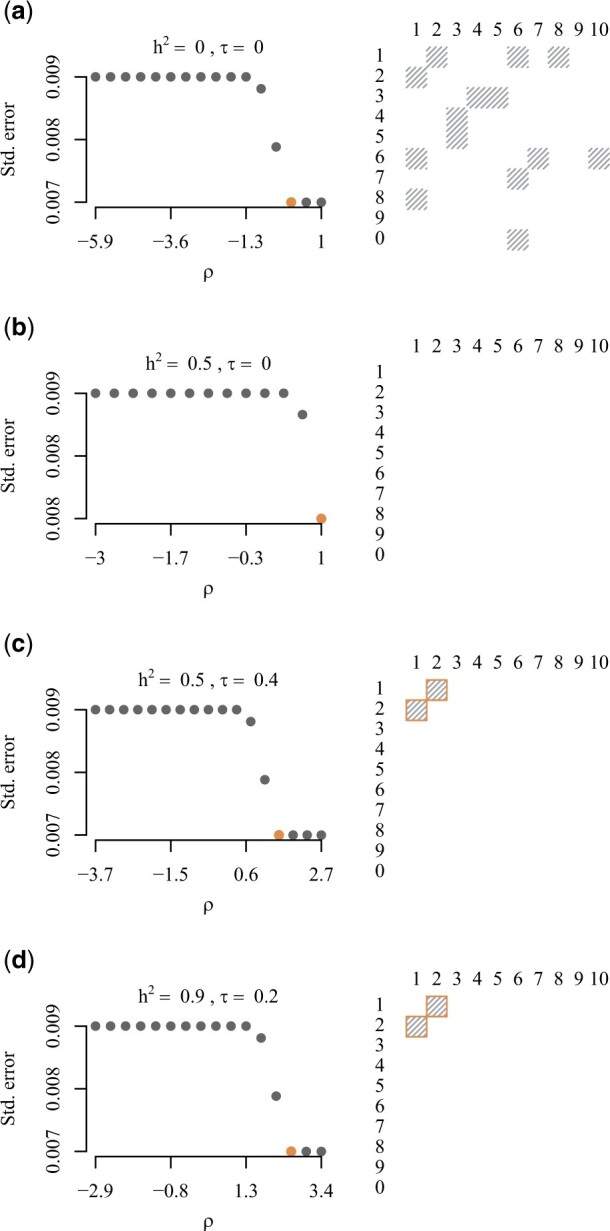
Diagnostics plots. *Left panel:* Average SE curve as a function of *ρ*. Orange dot indicates ${\hat{\rho }}_{\text{min}}$. *Right panel:* adjacency matrix corresponding to ${\hat{\rho }}_{\text{min}}$. Grey squares represent estimated edges, white space represents no edge, and orange borders represent the true edges.

In Fig. 1, we plot diagnostics for a randomly chosen simulation of different combinations of heritability and phenotypic correlation. In the left panel, we show the average SE averaged plotted against the values of *ρ* used in the fits. The orange-coloured dot indicates the minimum average SE and corresponding regularization parameter ${\hat{\rho }}_{\text{min}}$. In the right panel, we show a visualization of the estimated adjacency matrix for the clustering corresponding to ${\hat{\rho }}_{\text{min}}$ for one, randomly chosen, simulation. Grey squares represent estimated edges, white space represents no edges, and orange borders represent the true edges. In Fig. 1a, we observe that, in the case of no heritability and no phenotypic correlation, the average SE curve attains a minimal value (orange dot) yielding an somewhat empty adjacency matrix aligning with the truth of no clustered traits. In Fig. 1b, we observe that, in the case of $h_{1}^{2}=h_{2}^{2}=0.5$ and no phenotypic correlation, the average SE curve attains a minimal value (orange dot) yielding an almost empty adjacency matrix consistent with the truth of no clustered traits. In Fig. 1c and d, we observe that, for combinations of moderate to high heritability and phenotypic correlation, the minimal average SE is obtained exactly when the true clustering is discovered, as illustrated in the right panel.

**Figure 2.**
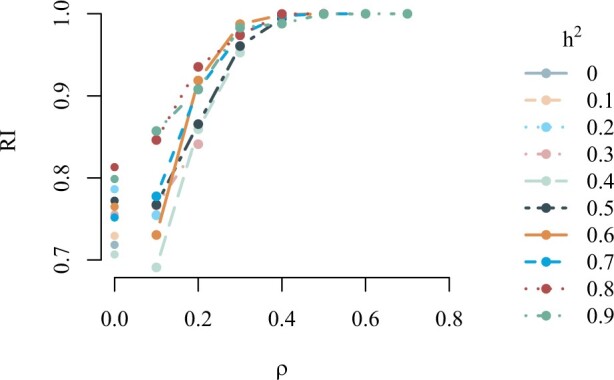
Rand index, RI, for the simulated scenarios at different values of the broad-sense heritability *h*^2^ and phenotypic correlation.

The sampling procedure is run with combinations of heritability and phenotypic correlation ranging between 0 and 0.9. The resulting average RIs are shown in Fig. 2. We observe that for a phenotypic correlation greater than 0.2, the RIs are greater than 0.9 regardless of the level of heritability, and when phenotypic correlation is greater than or equal to 0.4, the RIs are approximately 1, i.e. the true clustering is discovered in almost all of the 100 simulations.

**Figure 3.**
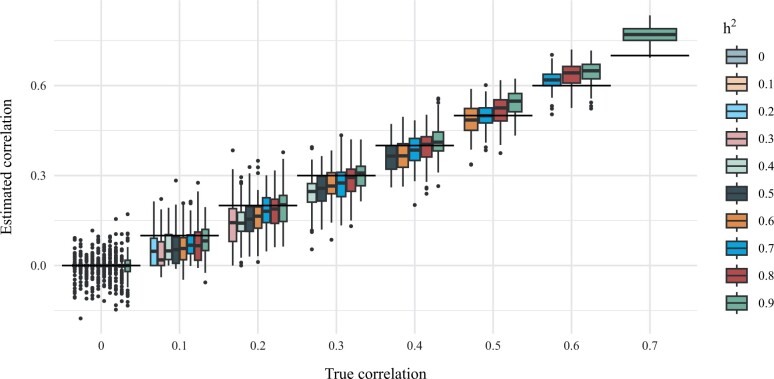
Comparisons of true phenotypic correlation and correlation estimation using geneJAM. The genetic correlation between $Y_{1}$ and $Y_{2}$ is demonstrated by a box plot which shows the quantiles of the estimates. The horizontal lines represent true values.

In Fig. 3, we show comparisons of the true phenotypic correlation and the correlation estimated by geneJAM by means of box plots. We observe that when the true phenotypic correlation is 0.5, the method provides unbiased estimates for phenotypic correlation for all levels of heritability. When the true phenotypic correlation is < 0.5, the method tends to slightly underestimate and when the genetic correlation is > 0.5 the method tends to slightly overestimate the phenotypic correlation. Furthermore, the smaller the heritability, the more the method underestimates and conversely for larger heritability. Finally, it should be noted that in the case where there is no phenotypic correlation in the data-generating process, the box plots are so flat as to appear non-existent.

As mentioned, a popular approach for estimating genetic correlation from summary statistics is cross-trait LD Score regression (Bulik-Sullivan *et al.* 2015a). The method relies on the fact that the GWAS effect size estimate for a given SNP incorporates the effects of all SNPs in linkage disequilibrium (LD) with that SNP (Bulik-Sullivan *et al.* 2015b; Yang *et al.* 2011b). In comparison with the geneJAM method, LD Score regression is more restrictive since it estimates pairwise correlations, while the geneJAM method estimates the regularized partial correlation.

We use cross-trait LD Score regression as reference to assess the performance of our method in terms of estimation of genetic correlation. We apply the method to simulated data described above. The genio package (Ochoa 2023) is used to convert the genotype and phenotype data into binary Plink format (.bed, .bim, .fam) file sets, and we use the recommended settings in the software package LDSC (v1.0.1) (Bulik-Sullivan *et al.* 2015b) to obtain LD Scores. The simple linear regression analyses are done in R using the MESS package (Ekstrøm 2019).

**Figure 4.**
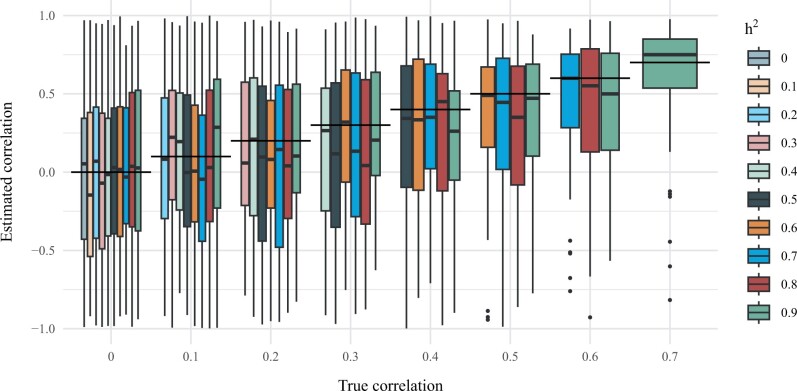
Comparisons of true phenotypic correlation and correlation estimation using geneJAM. The genetic correlation between $Y_{1}$ and $Y_{2}$ is demonstrated by a box plot which shows the quantiles of the estimates. The horizontal lines represent true values.

In Fig. 4, we show comparisons of the true phenotypic correlation and the correlation estimated by LD Score regression by means of box plots. We observe that while the method provides approximately unbiased estimates for the phenotypic correlation for all levels of heritability, the variability in the estimates is substantial.

We note that the model (6) assumes i.i.d. environmental errors across traits which may be unrealistic in practice. Assuming that environmental effects are not independent between traits, the covariance between traits *l* and l′ is

$$\text{cov}\left(C_{l},C_{l\prime }\right)=\rho_{ll\prime }\frac{V(X)V(C_{l})\;+\;\text{cov}(E_{l},E_{l\prime })}{E(X^{2})}.$$

**Figure 5.**
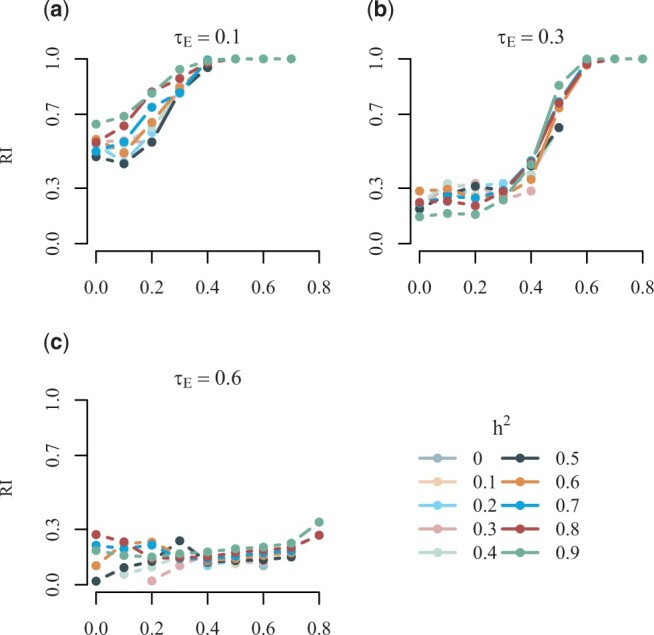
Rand index, RI, for the simulated scenarios at different values of the broad-sense heritability *h*^2^, phenotypic correlation, and correlation attributable to non-genetic effects.

**Figure 6.**
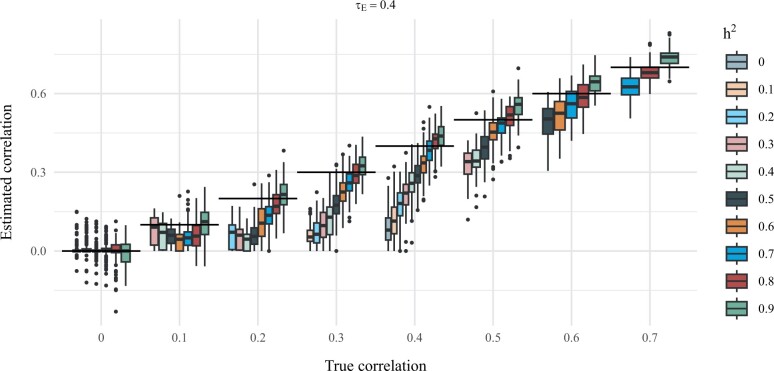
Comparisons of true phenotypic correlation and phenotypic correlation estimated using geneJAM. The phenotypic correlation between $Y_{1}$ and $Y_{2}$ is demonstrated by a box plot which shows the quantiles of the estimates. The horizontal lines represent true values. The results are stratified on values of the levels of correlation attributable to non-genetic effects.

To assess the clustering ability of geneJAM in this scenario, we consider, again, heritability from 0 to 0.9 and the values of phenotypic correlation are set from 0 to 0.8. The correlation attributable to non-genetic effects is also tried at 0 to 0.8. Thorough presentations are found in the Supplementary Material; here, we summarize our findings. The resulting average RIs are shown in Fig. 5. We observe that, overall, the larger the proportion of correlation *τ_E_* attributable to non-genetic effects the lower the RI. Specifically, for $\tau_{E}=0.1,$ (a) and a phenotypic correlation greater than 0.4, the RIs are approximately 1, i.e. the true clustering is discovered in almost all of the 100 simulations. For $\tau_{E}=0.3,$ (b), the phenotypic correlation should be greater than 0.6 for the RIs to be approximately 1, and for $\tau_{E}=0.6$ (c; and all $\tau_{E}\geq 0.6$), the RIs are for all levels of phenotypic correlation less than 0.4, i.e. the true clustering is discovered in less than 40 out of the 100 simulations. In Fig. 6, we show comparisons of the true phenotypic correlation and the correlation estimated by geneJAM. We observe that when the true phenotypic correlation is 0, the method provides unbiased estimates for phenotypic correlation for all levels of heritability. When the true phenotypic correlation is > 0, the method tends to underestimate but decreasingly so when the heritability increases. Comparing across levels of environmental correlation *τ_E_*, we see that the larger the portion of correlation being due to environmental factors, the more substantial the underestimation.

#### 3.1.3 Precision and computing time assessment

In this section, we compare the performance in terms of *precision* and *computing time* of the geneJAM, simple univariate linear regressions, and linear mixed effects models.

As mentioned, standard multiple linear regression models assume that the underlying observations are independent. As an extension to the multiple linear regression models *multilevel models* or *linear mixed-effects models* (LMM) assume observations to be inhomogeneous in the sense that they are not independent but grouped. If we treat multiple simultaneously measured traits **Y** in a repeated measurement setting, then a univariate multilevel model can be used to explain the phenotypic variation: We define $\overset{ˇ}{Y}=$$\text{vec}\left(Y\right)={\left(y_{1},\ldots ,y_{q}\right)}^{⊤}\in R^{Nq}$ as the column-wise vectorization of **Y**, i.e. $\overset{ˇ}{Y}={\left(y_{11},y_{21},\ldots ,y_{N1},y_{12},y_{22},\ldots ,y_{N2},\ldots ,y_{1q},y_{2q},\ldots ,y_{Nq}\right)}^{⊤},$ not to be mistaken with the row-wise vectorization of **Y** denoted by $\vec{Y}$ and introduced in (3). Then, the *univariate multilevel model* takes the form:

(7) $$\overset{ˇ}{Y}=\overset{ˇ}{Z}b\;+\;U\alpha +\overset{ˇ}{E},$$

where $\overset{ˇ}{Z}\in R^{Nq\times 2q}$ is a fixed-effects design matrix, $b\in R^{2q}$ is a vector of regression coefficients for the fixed effects, such that each trait has a fixed intercept and slope, $\alpha \in R^{N}$ are person-specific regression coefficients for the random effect with random-effects design matrix $U\in R^{Nq\times N}$, ensuring that each individual has its own trait-specific random intercept, and $\overset{ˇ}{E}=\text{vec}\left(E\right)={\left(e_{1},\ldots ,e_{q}\right)}^{⊤}\in R^{Nq}$ are trait-specific residual errors caused by non-additive genetic variation, random environmental effects, and measurement error. We define the random-effects design matrix as an identifier of the individuals and assume that

-
$e_{l}∼N_{N}(0,\sigma_{e}^{2}I_{N})$ and uncorrelated for l=1,…,q;
-
$\alpha ∼N_{N}(0,\sigma_{\alpha }^{2}G)$ ;
-
$e_{1},\ldots ,e_{q}$ , and α are independent.

Here $G\in R^{N\times N}$ is a general covariance matrix. Possible structures for **G** may be a block diagonal, a diagonal or a general positive definite matrix. This way, restricted maximum likelihood (REML) estimates of the parameters in models on the form (7) can be determined using the lmer function in the lme4 package for R (Bates *et al.* 2015). We assume a common correlation among the observations from a single individual, with the correlation being the same for all individuals. Compared to the assumptions for linear regression model (3) of the geneJAM method, the assumption is analogous to the residual covariance matrix Ω being a block diagonal with blocks corresponding to the individuals and with each block having a compound-symmetry structure. This structure has two unknown parameters: one modelling a common covariance and the other a residual variance. The form for **R** would then be as follows:

(8) $$R=\left(\begin{array}{cccc} \sigma_{e}^{2}+\sigma_{\alpha }^{2} & \sigma_{e}^{2} & \cdots & \sigma_{e}^{2}\\ \sigma_{e}^{2} & \sigma_{e}^{2}+\sigma_{\alpha }^{2} & \ddots & \vdots \\ \vdots & \ddots & \sigma_{e}^{2}+\sigma_{\alpha }^{2} & \vdots \\ \sigma_{e}^{2} & \cdots & \sigma_{e}^{2} & \sigma_{e}^{2}+\sigma_{\alpha }^{2} \end{array}\right).$$

In comparison with the geneJAM method, this is more restrictive as the geneJAM method allows for an arbitrary structure of the residual covariances **R** among traits within each individual in (4).

We use this method as reference when assessing the precision and computational performance of our method. We also compare to fitting simple linear regression models for each trait and corresponding PGS separately.

We simulate 100 data sets according to the sampling procedure described in subsubsection 3.1.1.

**Figure 7.**
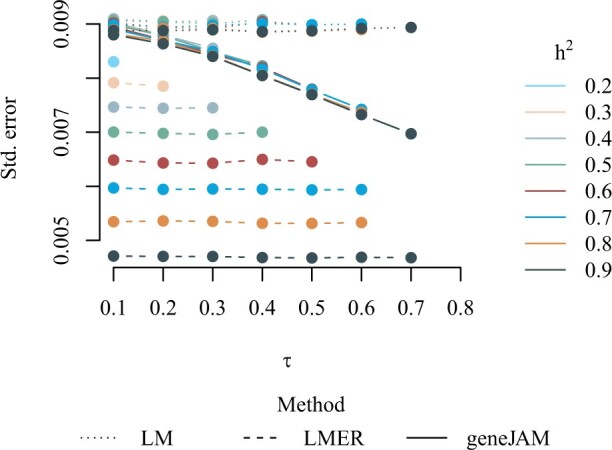
Standard error of the estimates in the active set averaged over 100 simulations with different levels of heritability and phenotypic correlation.

We are interested in improving the precision of the estimates of traits on which there are genetic effects. We refer to this set of estimates as the *active set*. In Fig. 7, we show the standard error of the estimates in the active set averaged over the 100 simulations. We observe that regardless of the scenario and heritability the geneJAM performs better than the simple linear regression (LM) while the multilevel model (LMER) yields the best absolute precision for all tried combinations of heritability and phenotypic correlation. As expected, the larger the phenotypic correlation, the better the absolute precision of the geneJAM, while, for a certain level of phenotypic correlation, the level of heritability does not affect the precision notably. This contrasts the precision of the LMER which is approximately constant in *τ*, while it decreases when *h*^2^ increases.

**Figure 8.**
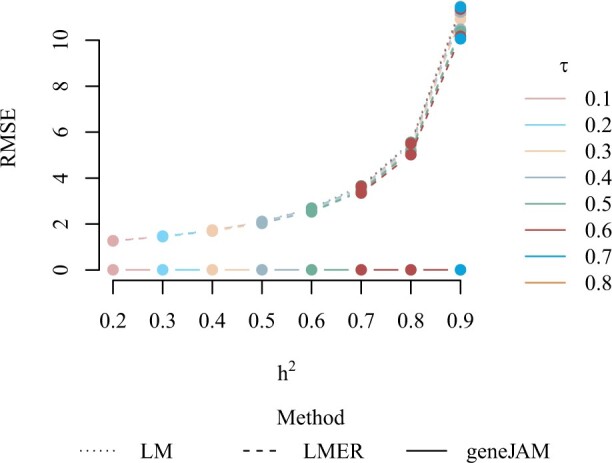
Root mean squared error (RMSE) of the estimates in the active set averaged over 100 simulations with different levels of heritability and phenotypic correlation.

In Fig. 8, we show the RMSE in the active set averaged over the 100 simulations. The figure shows that, in general, the prediction performance of the methods discussed is constant in the phenotypic correlation. As expected, the RMSE is the same for the LM and LMER methods. The geneJAM is also constant in the heritability, while the LMER and LM are worsening when the heritability grows since the RMSE is approximately exponentially increasing in *H*^2^. For all levels and combinations of heritability and phenotypic correlation, the geneJAM method clearly outperforms the other methods in terms of predictive ability.

**Figure 9.**
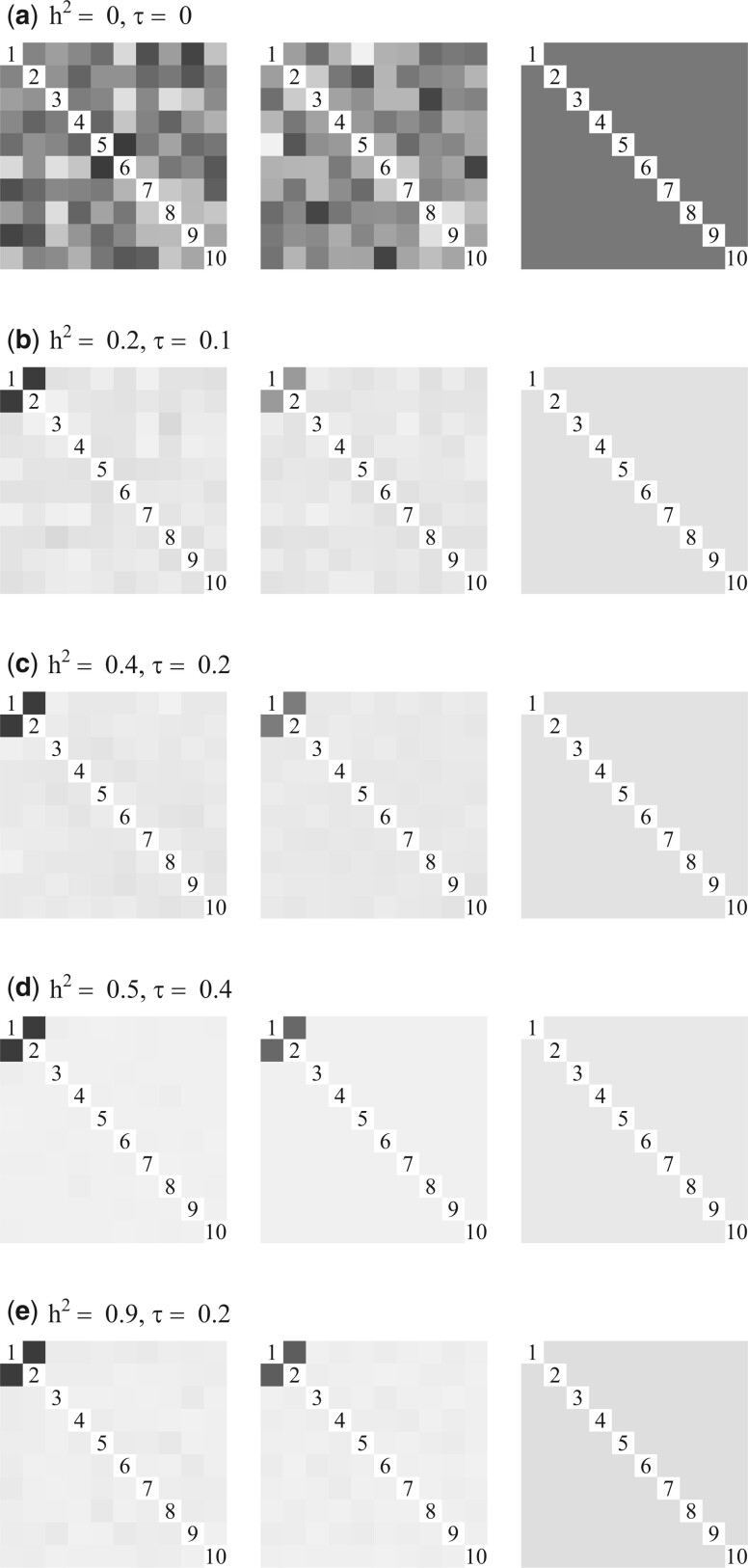
Diagnostics plots. *Left panel:* Average SE curve as a function of *ρ*. Orange dot indicates ${\hat{\rho }}_{\text{min}}$. *Right panel:* adjacency matrix corresponding to ${\hat{\rho }}_{\text{min}}$. Grey squares represent estimated edges, white space represents no edge, and orange borders represent the true edges.

In Fig. 9, we visualize the covariance structure between traits for simulations with different combinations of level of heritability and phenotypic correlation. Each row (a)–(e) corresponds to a specific combination. Dark colours indicate large values, and light colours indicate small values. In the left panel, we show the structure induced by the sampling, in the centre panel we show the structure estimated by the geneJAM method, and in the right panel we show the structure estimated by the multilevel model. We observe that, whenever the phenotypic correlation is ≥0.1, the geneJAM method appears to successfully capture the covariance structure induced by the sampling. This is not possible for the multilevel model which requires a fixed structure such as (8).

To assess the computation time of the geneJAM, we consider a simulation design similar to the one proposed in the previous subsection with a phenotypic correlation of 0.5 and broad-sense heritability $H^{2}=0.8$. We assume that the grouping is given, so we do not run the graphical lasso part of the procedure. To assess the performance of the R code, we use the package microbenchmark by Mersmann (2019) on an AMD EPYC 7702P 64-Core Processor.

**Figure 10.**
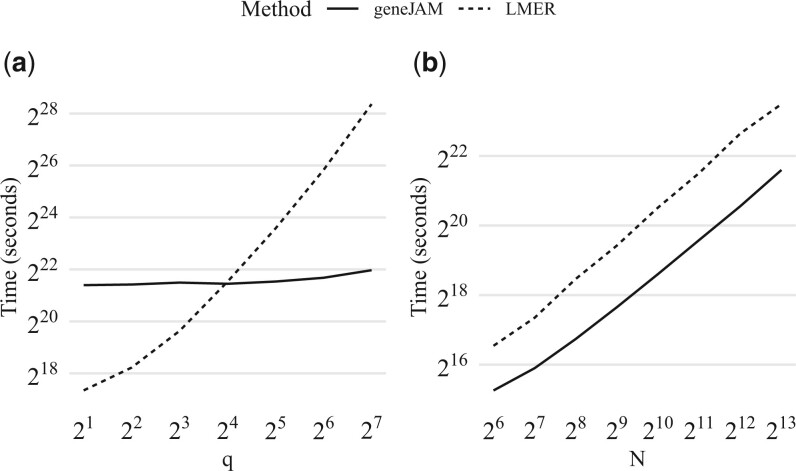
Scaling (measured in seconds) of the geneJAM (solid) and multilevel model (dashed) in the number of traits *q* (a) and observations *N* (b).

In Fig. 10, we show how the geneJAM (solid) and multilevel model (dashed) scale in execution time in the number of traits *q* (a) and observations *N* (b). To assess the scaling in the number of traits each sample is generated with N≃8000 observations. First, we observe that the computing time of the geneJAM is almost constant in *q*. Second, we observe that while the mixed model is faster than the geneJAM for *q *<* *15, the computing time increases almost exponentially as the number of traits increases logarithmically. For *q *>* *15, the mixed model is slower than geneJAM, and the lme4 cannot handle $q>2^{7}$. To assess the scaling in the number of observations, each sample is generated with *q *=* *32 traits. As expected, given the number of traits fixed at 32, the geneJAM is for all values of *N* faster than the multilevel model. The computing time increases similarly for the two methods.

To assess the memory usage of the geneJAM, we consider a simulation design similar to the one above and use the package pryr by Wickham (2023) on an AMD EPYC 7702P 64-Core Processor.

**Figure 11.**
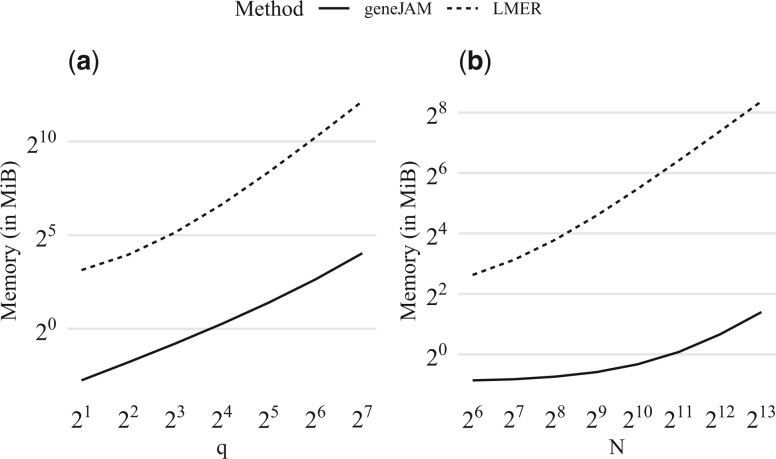
Scaling (measured in MiB) of the geneJAM (solid) and multilevel model (dashed) in the number of traits *q* (a) and observations *N* (b).

In Fig. 11, we show how the geneJAM (solid) and multilevel model (dashed) scale in memory usage in the number of traits *q* (a) and observations *N* (b). Please note that both axes are scaled by log base 2. To assess the scaling in the number of traits, each sample is generated with $N=2^{13}≃8000$ observations. We observe that while the memory usage for both methods increases approximately linearly in *q*, the geneJAM is consistently approximately a factor 2^5^ more memory efficient than the multilevel model. To assess the scaling in the number of observations, each sample is generated with *q *=* *32 traits. Given the number of traits fixed at 32, the memory usage for both methods increases in *N.* The geneJAM is consistently (and increasingly in *N*) less memory consuming than the multilevel model.

### 3.2 Real data

We apply geneJAM to the heterogeneous stock of mice data set available from the Wellcome Trust Centre for Human Genetics (Flint and Valdar 2008).

The heterogeneous stock of mice consists of 1904 individuals from 85 families, all descended from eight inbred progenitor strains (Valdar *et al.* 2006). We consider this particular data set not only because it contains multiple quantitative traits, but also because many of the traits are naturally associated, and thus this data set presents a realistic mix between our simulation scenarios. Please note that the aim of the example is to demonstrate proof of concept; the proposed method is used as a screening tool for identifying potential clusters of the traits. This way we can subsequently investigate the associations using more complicated nested models taking into account the biological relationships of related subjects. The data contain 129 quantitative traits which are classified into six broad categories including behaviour, diabetes, asthma, immunology, haematology, and biochemistry. Because of the fairly large amount of missing data in the traits, and to avoid having to discuss complicated imputation techniques, we narrow down the analysis and include only the 14 traits in the asthma category which has the fewest missing values. Development and deployment of a phenotyping protocol to collect measures on a model of asthma is described in Solberg *et al.* (2006) as well as a definition of the 14 traits in the asthma category. We omit individuals with missing trait and obtain *N *=* *1491 observations. A total of 12 226 autosomal SNPs were available for all mice. As Crawford *et al.* (2018), for “individuals with missing genotypes, we imputed missing values by the mean genotype of that SNP in their family. All polymorphic SNPs with minor allele frequency above 1% in the training data were used for prediction”. Furthermore, SNPs with no variation across observations are removed and we obtain *p *=* *10 994 SNPs.

We are interested in clustering traits sharing genetic characteristics in preparation for fitting low-dimensional multivariate linear regression models. For each quantitative trait, we generate a PGS as described in subsection 2.1. on the entire data set. GeneJAM is applied at a suitable sequence of the regularization parameters *ρ*.

**Figure 12.**
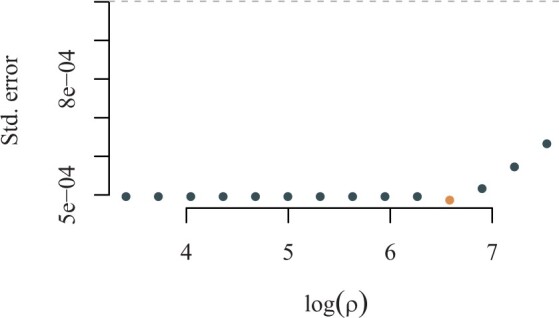
Diagnostics plot for the heterogeneous stock of mice data set: average SE curve as a function of *ρ*.

**Figure 13.**
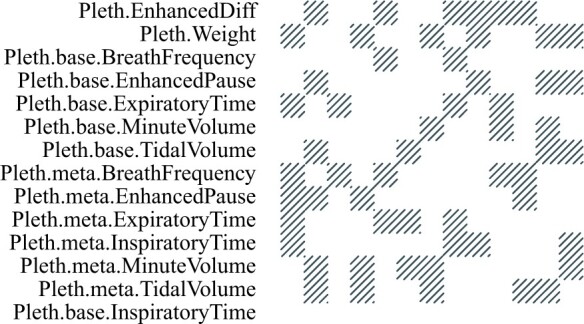
Diagnostics plot for the heterogeneous stock of mice data set: adjacency matrix corresponding to ${\hat{\rho }}_{\text{min}}$. Grey squares represent estimated edges and white spaces represent no edges.

In Figs 12 and 13, we show diagnostics plots. In the top row, we show the average SE plotted against the values of *ρ* used in the fits. The orange-coloured dot indicates the minimum average SE and corresponding regularization parameter ${\hat{\rho }}_{\text{min}}$. We observe that the average SE curve attains a minimum at ${\hat{\rho }}_{\text{min}}$. In the bottom row, we show a visualization of the estimated adjacency matrix for the clustering corresponding to ${\hat{\rho }}_{\text{min}}$. Grey squares represent estimated edges, and white space represents no edges. We observe that the best precision is obtained when all traits except for one are clustered together. The singleton cluster identified is the sixth trait (Pleth.base.InspiratoryTime) which is defined as the baseline inspiratory time measured by the plethysmograph (Solberg *et al.* 2006).

**Figure 14.**
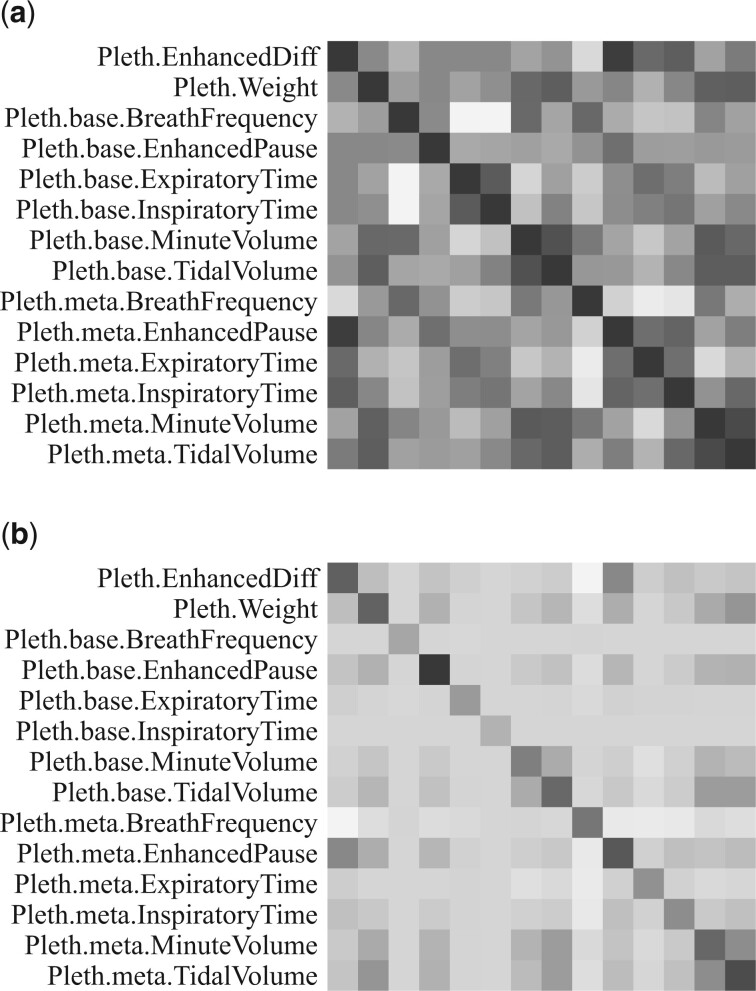
Visualization of the covariance structure for the heterogeneous stock of mice data set. Dark colours indicate large values, and light colours indicate small values. (a) Structure observed in the data. (b) Structure estimated by the geneJAM method.

In Fig. 14, we visualize the covariance structure between traits for the heterogeneous stock of mice data set. Dark colours indicate large values, and light colours indicate small values. In the top row, we show the structure observed in data by estimating the pairwise covariances between traits, and in the bottom row we show the structure estimated by the geneJAM method. We observe some similar structures in the two figures, e.g. a stronger correlation between Pleth.meta.MinuteVolume and Pleth.meta.TidalVolume and between Pleth.base.MinuteVolume and Pleth.base.TidalVolume. This is indication of two pairs of genetic relationships among the traits. We also see structures in the observed covariance which is not present in the structure estimated by the geneJAM method. There is, e.g. a stronger observed relationship between Pleth.meta.EnhancedPause, Pleth.meta.ExpiratoryTime, and Pleth.meta.InspiratoryTime. This may be indication of traits that are related for other reasons than genetics and the relations are, therefore, not captured by the geneJAM method.

## 4 Discussion

In this paper, we have proposed a method, which uses PGSs and graphical lasso for clustering simultaneously measured traits sharing genetic characteristics in preparation for fitting low-dimensional multivariate linear regression models. Furthermore, we have proposed a procedure for choosing the optimal regularization for the graphical lasso as well as a method for fitting the models using the FGLS estimator.

Key advantages of our framework are that there are no prior assumptions on the structure or sizes of the clusters of traits, information on PGS level is enough when data on SNP level are not available, and, following the clustering and modelling, both clusters and model fit are readily available. Moreover, the computing time is highly satisfactory even for large data, and compared to both the simple linear regression and the multilevel models the geneJAM is superior in terms of precision of the estimates of traits on which there are genetic effects. Finally, if (independent) environmental factors are available, they are easily adjusted for.

To understand the relevance of cluster assignment, we simulated data sets with different cross-trait structures and effect sizes. In the extreme case, where no traits are affected by the SNPs, the RI is close to one. Moreover, as expected, the estimated adjacency matrix quickly reaches the true zero matrix. In the extreme case, where all traits are equally affected by all SNPs, the geneJAM does not perform very well—as would be expected; the RI decreases as the heritability increases and even for small values, the RI is only 0.11. Similarly, the estimated adjacency matrix goes from the true one matrix to the zero matrix at which the minimum average SE is (erroneously) attained. More interestingly, when a subset of features affect a subset of clustered traits and when the effect sizes are sufficiently large, the average SE attains a maximum value (not at the tails of the sequence of *ρ*), indicating that a range of values of the regularization parameter exists, such that an optimal (attainable) agreement between the estimated and the true clustering is reached. This, indeed, appears to be the case, as indicated by the sequences of estimated adjacency matrices: in general, the sparsity of the matrix increases in *ρ*, and for a range of values of *ρ* the estimate is equivalent to the adjacency matrix representing the true clustering of traits. Furthermore, the larger the effect size, the larger the range at which the clustering is correctly estimated.

Moreover, using the implemented tuning method we are able to determine a value of *ρ* at which the true clustering is identified as the value at which the average SE is minimized.

In practice, researchers may have preconceived ideas about clusters of traits sharing genetic characteristics. If no clusters are identified by the geneJAM, it is because the genetic signal is too low or the presumption is wrong, i.e. the traits are clustered but the clustering is due to shared environment. If the traits are in fact not clustered, there is nothing to gain by a joint analysis of the traits. If the traits are clustered due to shared environment non-genetics effects, other more suitable methods are advised as multiple studies have shown that the joint analysis of multiple traits can achieve larger power as compared to single-trait analysis when the traits have shared environment but no shared genetics (e.g. Aschard *et al.* 2017). It should be emphasized that traits may be correlated due to other (non-genetic) factors, in which case the geneJAM may be unable to detect the clusters. If, however, summary statistics related to the correlation (e.g. metabolite risk scores) are available, these can be used in the method instead of PGSs to obtain clusters of traits sharing other (e.g. metabolic) characteristics.

While the multilevel model is useful where multiple correlated measurements are made on each individual, the geneJAM allows for correlation of traits, thereby improving the precision of the estimates of traits on which there are genetic effects. We intend to extend the geneJAM method to also allow for correlated measurements on each individual, to investigate the behaviour of related individuals. Numerous methods have been proposed for estimating the genetic relationships between individuals from SNPs. These may serve as inspiration for extension of the geneJAM method to related individuals. For example, Yang *et al.* (2011a) introduce a software tool called genome-wide complex trait analysis (GCTA). GCTA estimates the variance explained by all the SNPs on a chromosome or on the whole genome for a complex trait rather than testing the association of any particular SNP to the trait. However, currently GCTA does not include options to do multivariate analyses.

To put into perspective the ability of the geneJAM to estimate phenotypic correlation, we compared it to cross-trait LD Score regression. While the LD Score appears to yield an unbiased estimate of the phenotypic correlation, the variation in the estimates is substantial. In comparison with the geneJAM method, the LD score regression is more restrictive as it estimates only pairwise correlations. This may explain some of the variability. For both methods, it is true that to estimate the genetic overlap for many pairs of traits, measurement of multiple traits for the same individuals is required. Consequently, it is challenging to scale these designs to a large number of traits, in particular traits that are costly or in some way or another difficult to measure, such as low-prevalence diseases.

The algorithms presented in Zhou and Stephens (2014) are computationally efficient algorithms for fitting multivariate linear mixed models in GWASs. In practice, however, there could remain both computational and statistical barriers to applying the methods to even a quite modest number of traits, e.g. q≈10.

The geneJAM method relies on the assumption of multivariate normality. Within a homogenous population, PGSs based on a sum of independent variables (here SNPs) with identical distributions approximate a Gaussian distribution by the central limit theorem. However, the use of many correlated SNPs or a sample of heterogenous ancestry, i.e. SNPs with decidedly different genotype distributions, can lead to non-normal PGS distributions. Thus, inspection of PGS distributions may be called for in practical applications.

## Algorithmic overview

**Algorithm 1** geneJAM algorithm

**for** l=1,…,q **do**

  Compute ${\hat{b}}_{l}^{O}$ and ${\hat{u}}_{l}$

**end for**

Centre PGSs **Q** per column

Compute empirical covariance matrix Σ of centred **Q**

**if** no sequence of regularization parameters *ρ_r_*, r=1,…,R, is provided **then**

  Specify length *R* of sequence of regularization parameter *ρ_r_*

  Specify ratio *δ* between regularization parameters *ρ_r_*, r=1,…,R

  Choose a sequence of regularization parameters *ρ_r_*, r=1,…,R, with maximal value, *ρ_R_*, defined by the maximum column sum of Σ and the rest of the sequence determined by *R* and *δ*.

**end if**

**for** r=1,…,R **do**

  Estimate sparse precision matrices ${\hat{P}}^{\left(\rho_{r}\right)}$ to obtain $C^{\left(\rho_{r}\right)}$ clusters

  **for**$g(\rho )=1,\ldots ,C^{\left(\rho_{r}\right)}$**do**

    Compute ${\hat{\Omega }}_{g(\rho )}^{O}(\rho )$

  **end for**

  Construct ${\hat{\Omega }}^{O}$

  Compute estimate ${\hat{\vec{B}}}^{F}$

  Define ${\hat{\Omega }}^{F(1)}={\hat{\Omega }}^{O}$

  Initialize t→1

  **repeat**

    Compute ${\hat{u}}^{F(t)}$

    **for**$g(\rho )=1,\ldots ,C^{\left(\rho_{r}\right)}$**do**

      Compute ${\hat{\Omega }}_{g(\rho )}^{F(t)}$

    **end for**

    Construct ${\hat{\Omega }}^{F(t)}$

    Compute estimate ${\hat{\vec{B}}}^{F(t+1)}$

  **until** convergence

**end for**

## Supplementary Material

vbad192_Supplementary_Data

## Data Availability

GeneJAM is implemented in R (Buchardt 2022) and is available in the geneJAM repository at https://github.com/abuchardt/geneJAM. The simulated data underlying this article are also available at the repository. The real data underlying this article are available in WTCCC at http://mtweb.cs.ucl.ac.uk/mus/www/mouse/HS/index.shtml.
