# Supplementary material for: Joint regression analysis of multiple traits based on genetic relationships
Source: Bioinform Adv. 2024 Jan 4;4(1):vbad192. doi: 10.1093/bioadv/vbad192 (PMC10805347; doi:10.1093/bioadv/vbad192)
Supplement: vbad192_Supplementary_Data [file vbad192_supplementary_data.pdf]

Bioinformatics Advances, 2023, pp. 1–5

doi: DOI HERE
Advance Access Publication Date: Day Month Year
Paper

— Supplementary —

PAPER

Joint Regression Analysis of Multiple Traits Based on Genetic Relationships

Ann-Sophie Buchardt[1,*] Xiang Zhou[2]
and Claus Thorn Ekstrøm[3]

[1]Department of Public Health, University of Copenhagen, Øster Farimagsgade 5, 1014, Copenhagen, Denmark, [2]Department of Biostatistics, University of Michigan, 1415 Washington Heights, 48109, Michigan, United States and [3]Department of Public Health, University of Copenhagen, Øster Farimagsgade 5, 1014, Copenhagen, Denmark

*Ann-Sophie Buchardt. ann-sophie@buchardt.net
FOR PUBLISHER ONLY Received on 12 April 2023; revised on 12 December 2023; accepted on 15 December 2023

Abstract

Supplementary material.

Simulated data

Non-genetic correlation

In this section we present the result of applying the geneJAM method when environmental effects are not independent between traits. In the Figures 1–9 Comparisons of true phenotypic correlation and phenotypic correlation estimated using geneJAM. The phenotypic correlation between $\mathbf{Y}_1$ and $\mathbf{Y}_2$ is demonstrated by a box plot which shows the quantiles of the estimates. The horizontal lines represent true values. The results are stratified on values of the levels of correlation attributable to non-genetic effects.

© The Author 2023. Published by Oxford University Press. All rights reserved. For permissions, please e-mail: journals.permissions@oup.com

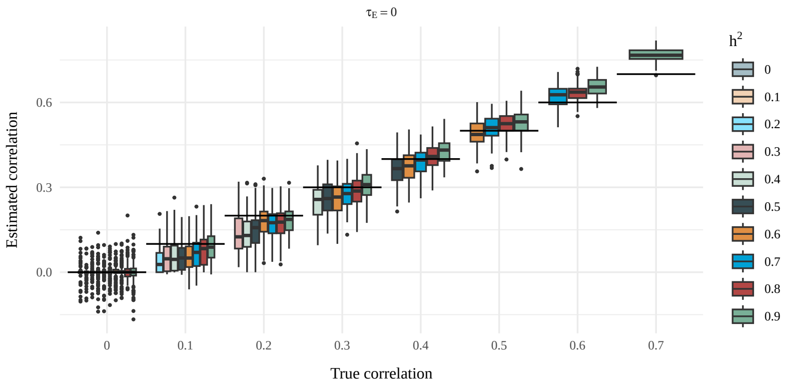

Fig. 1.

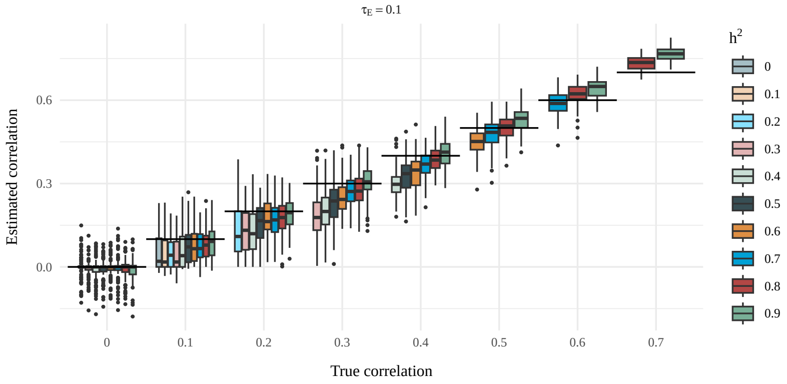

Fig. 2.

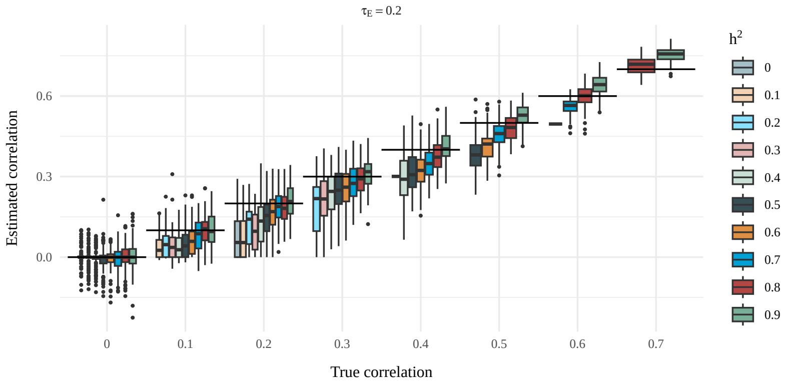

Fig. 3.

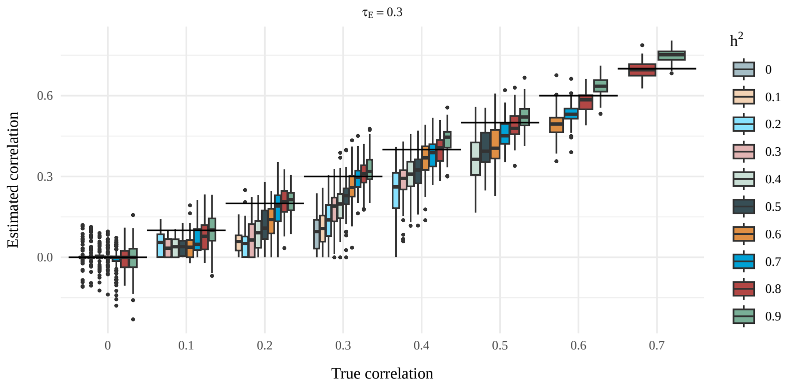

Fig. 4.

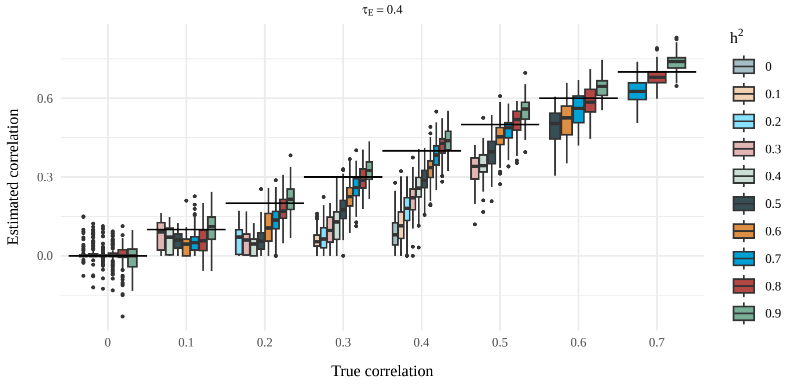

Fig. 5.

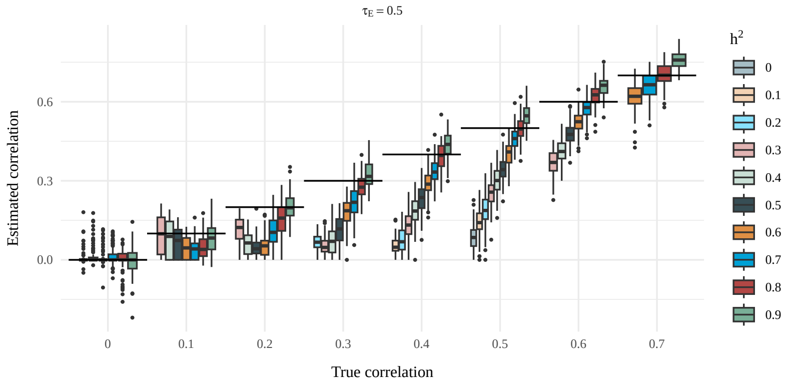

Fig. 6.

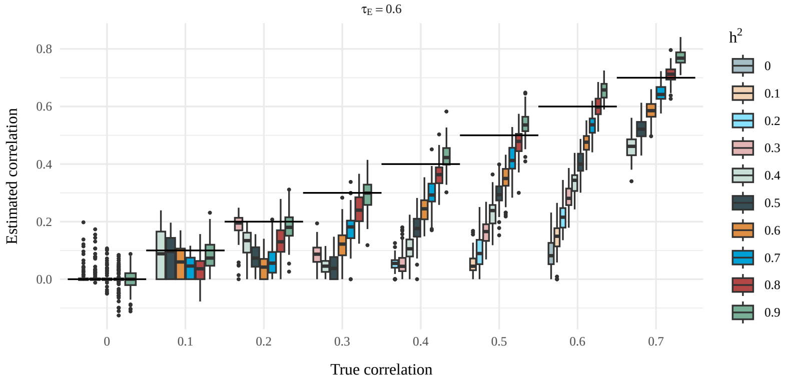

Fig. 7.

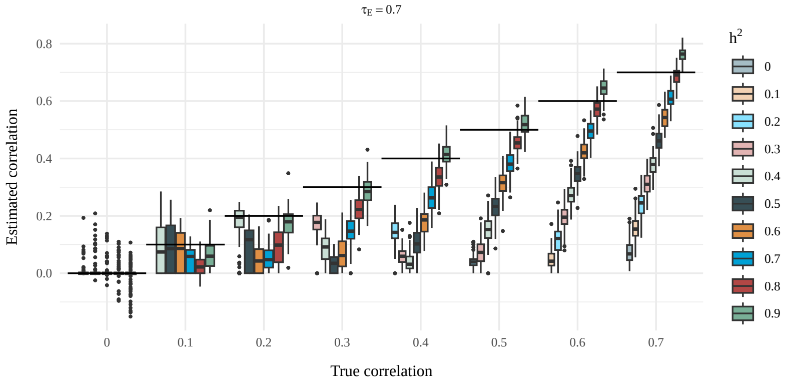

Fig. 8.

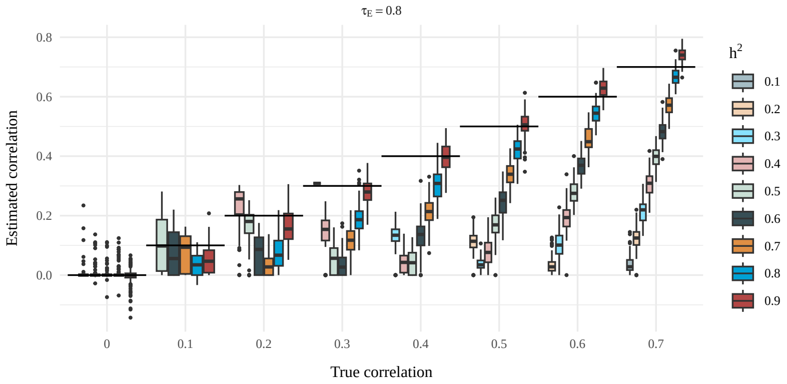

Fig. 9.

Genetic correlation – estimated adjacency

In this section we present the result of applying the geneJAM method on all viable combinations of (tried) heritability and phenotypic correlation.

In Figures 10–53 we show visualisations of the estimated adjacency matrices at all values of the sequence of ρ tried. Grey squares represent estimated edges, white space represents no edges, and orange borders represent the true edges. The matrix surrounded by a black border represents the matrix attained at the the minimum average SE. As expected, the sparsity of the matrix increases in ρ, and when the heritability and/or the phenotypic correlation is large enough the estimated adjacency matrix is, for a range of values of ρ, equivalent to the adjacency matrix representing the true clustering of traits.

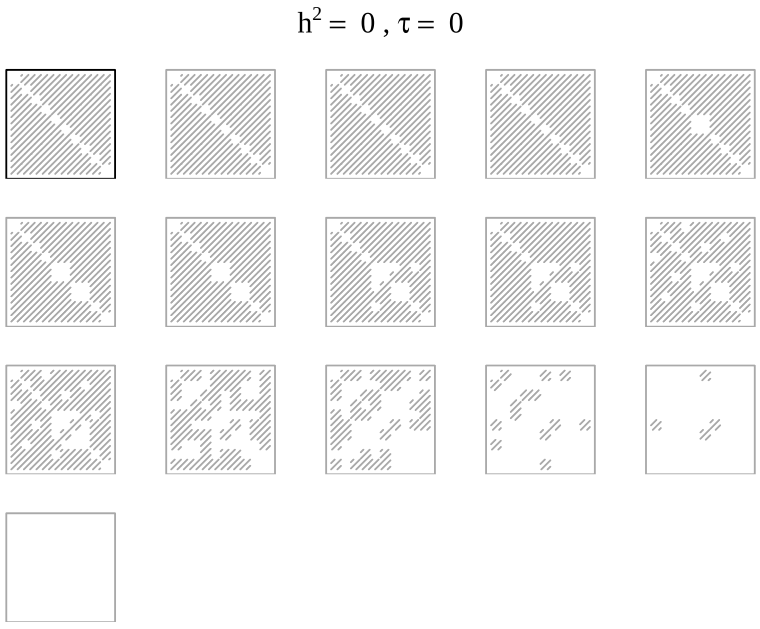

Fig. 10.

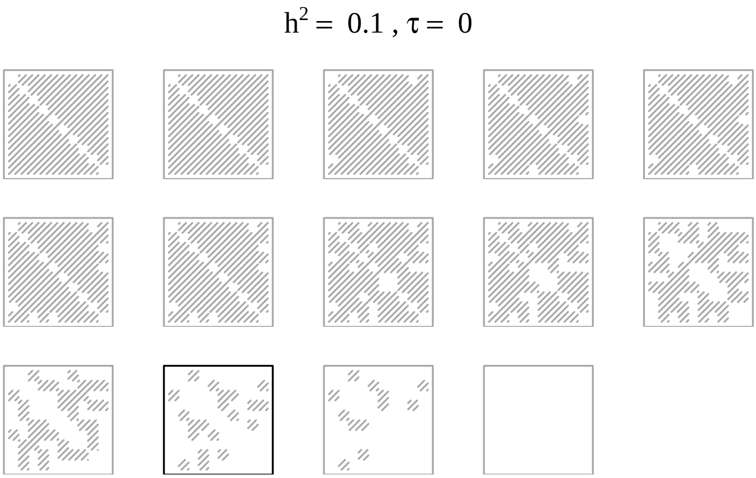

Fig. 11.

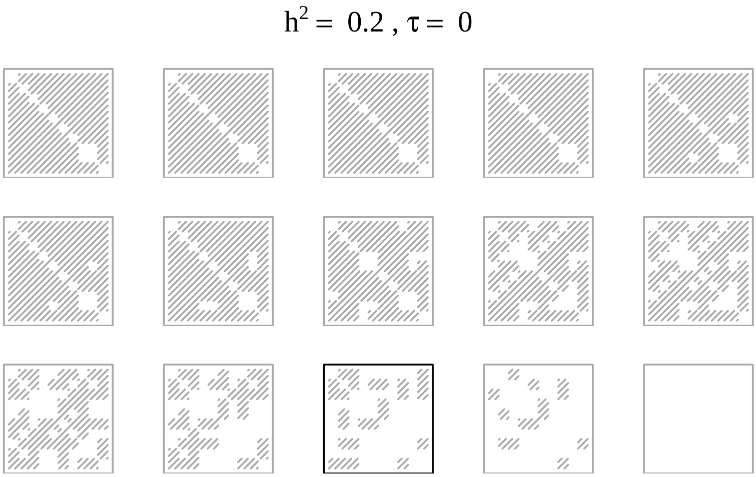

Fig. 12.

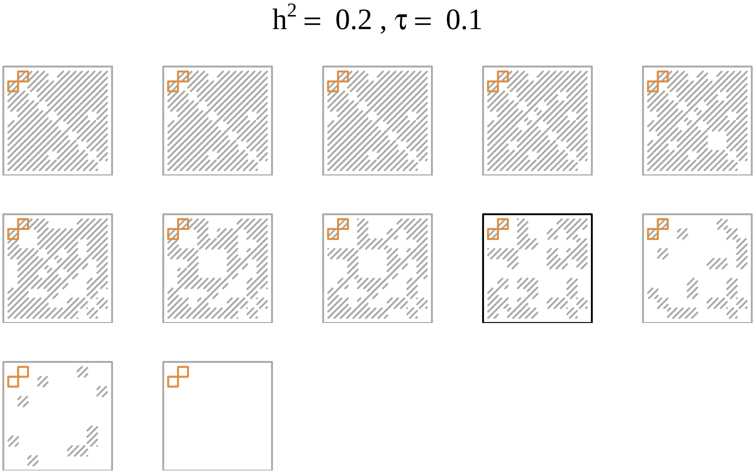

Fig. 13.

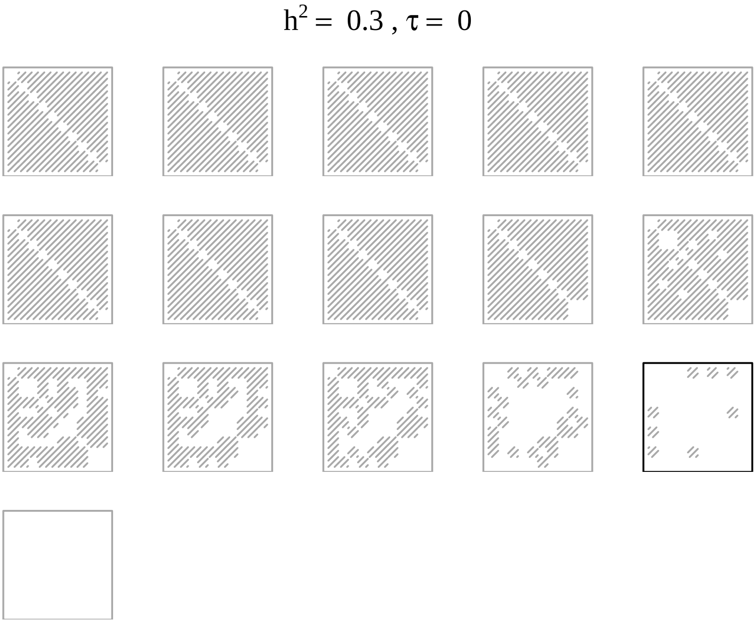

Fig. 14.

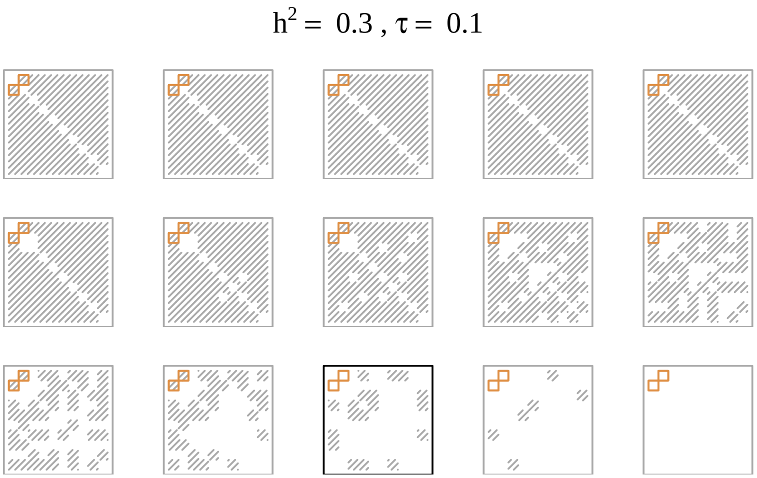

Fig. 15.

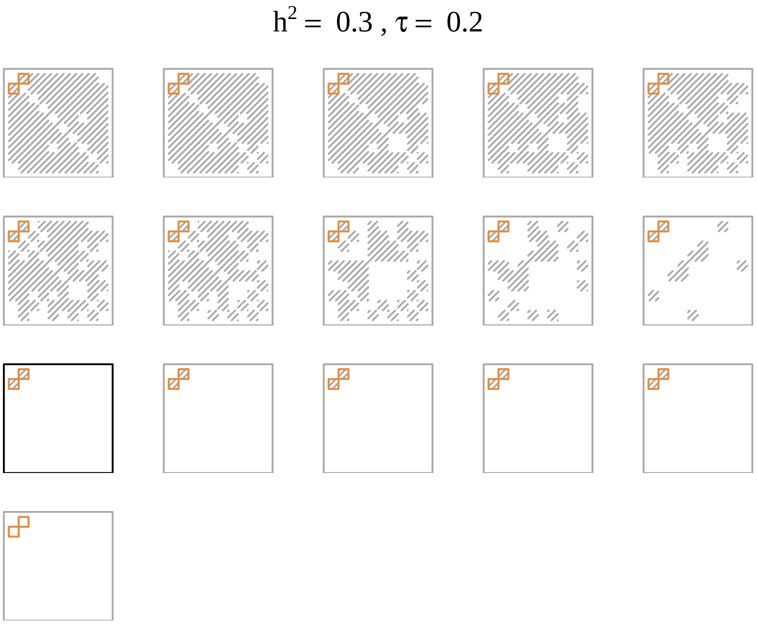

Fig. 16.

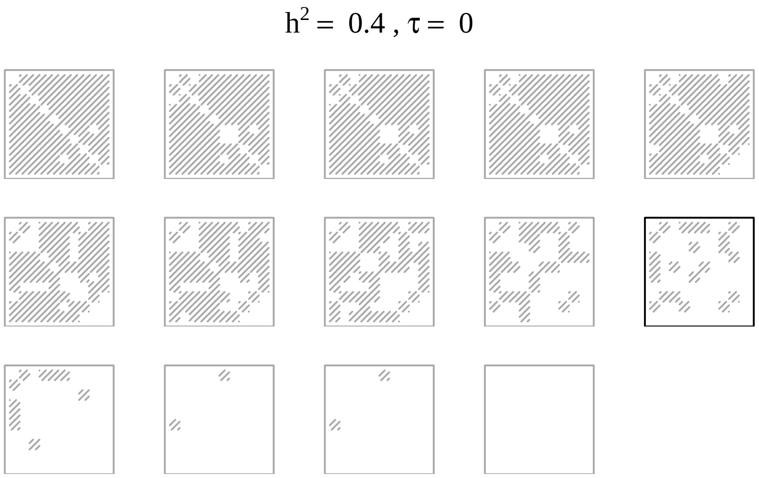

Fig. 17.

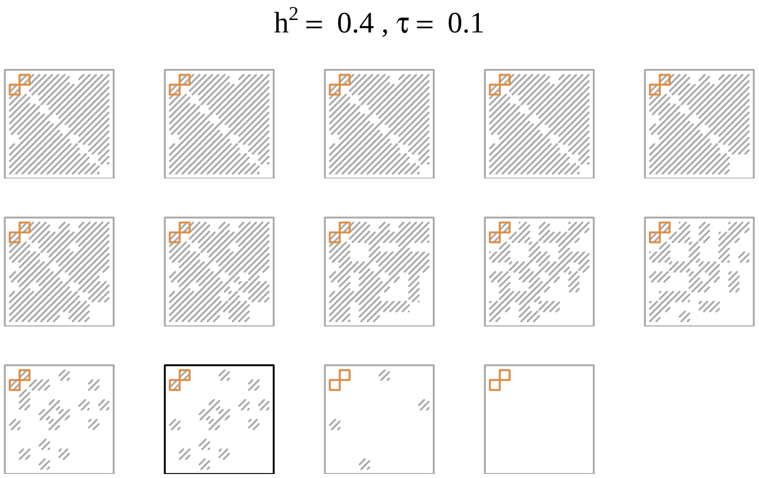

Fig. 18.

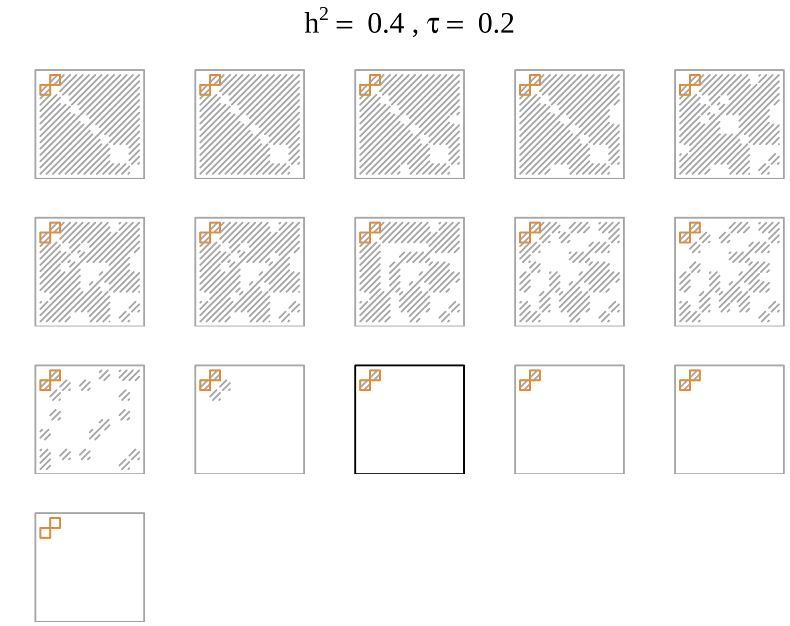

Fig. 19.

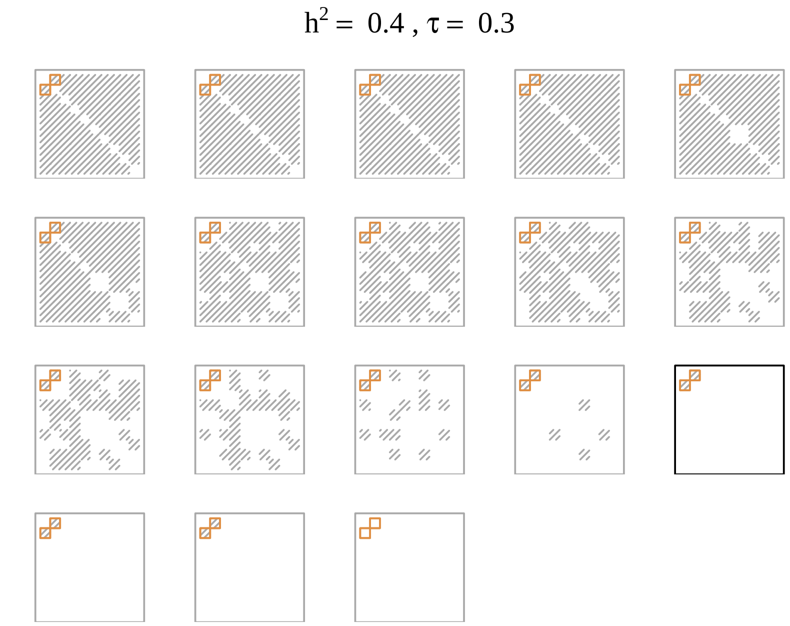

Fig. 20.

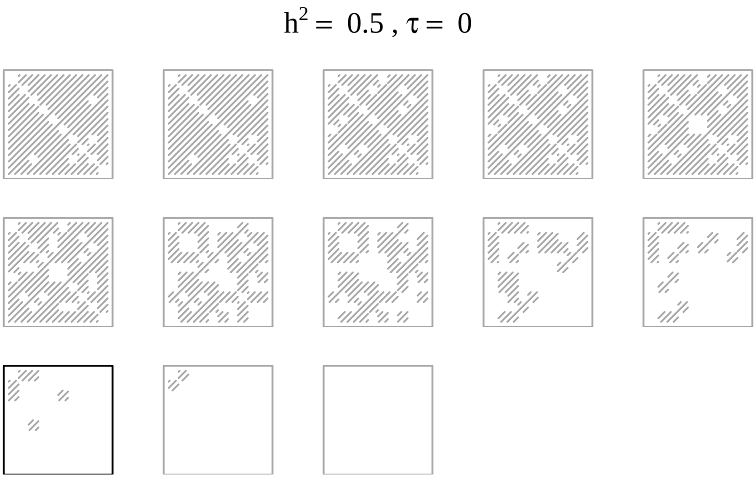

Fig. 21.

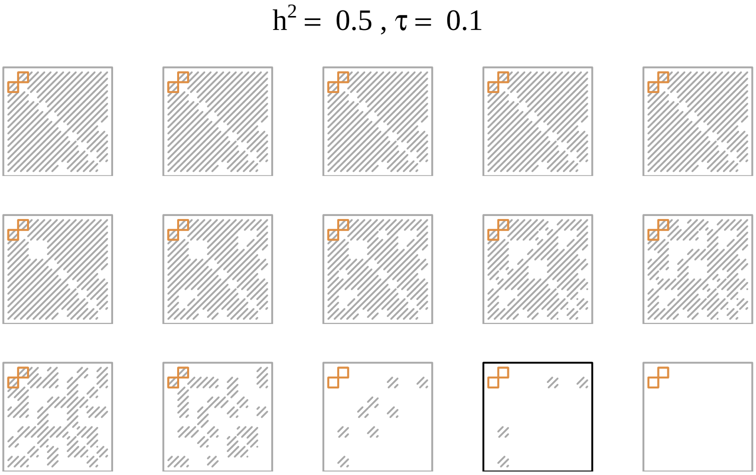

Fig. 22.

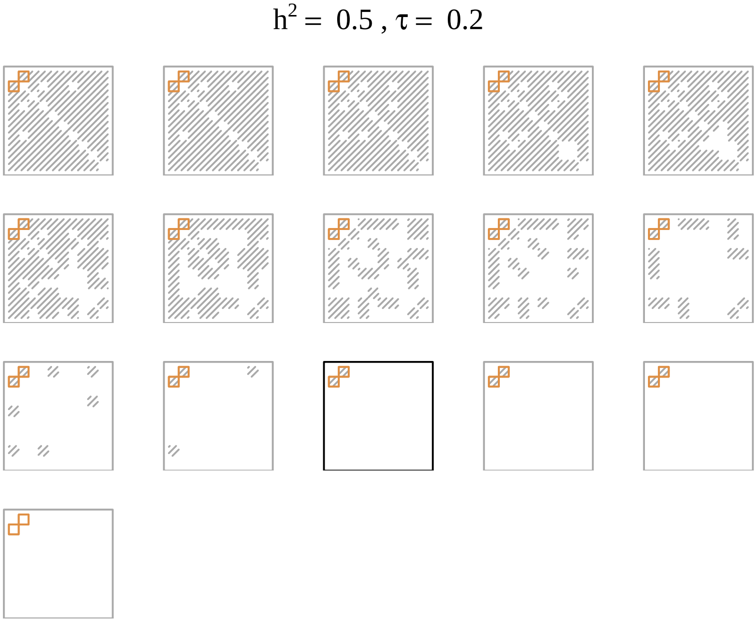

Fig. 23.

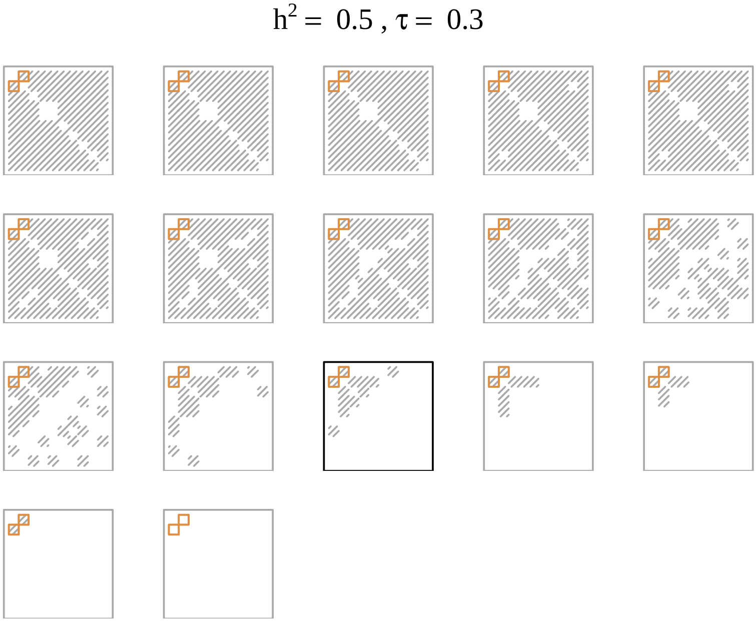

Fig. 24.

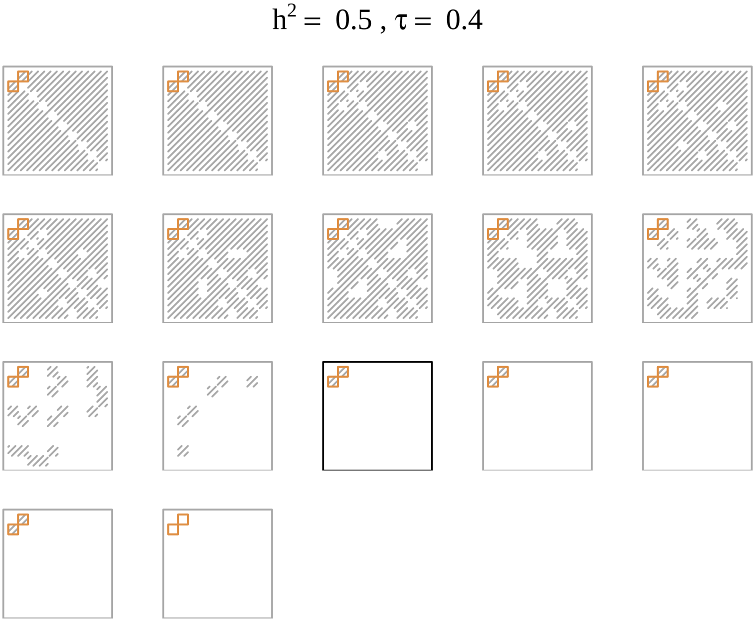

Fig. 25.

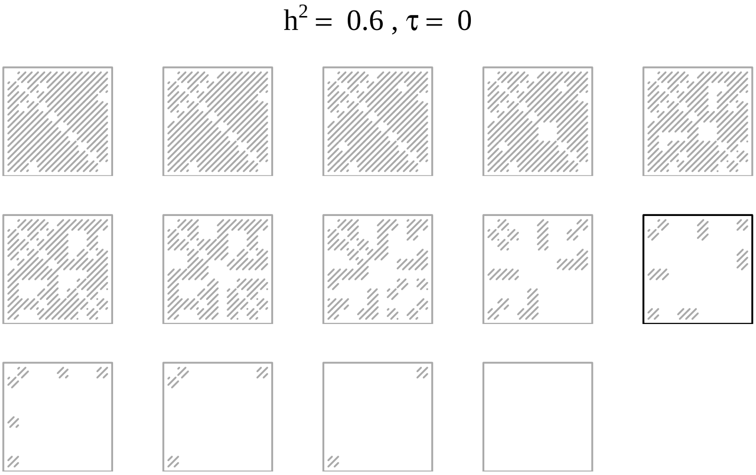

Fig. 26.

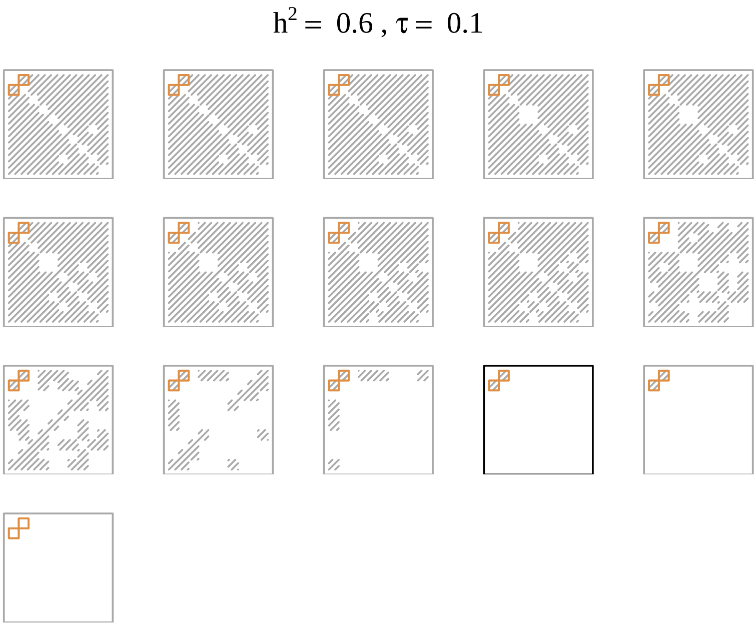

Fig. 27.

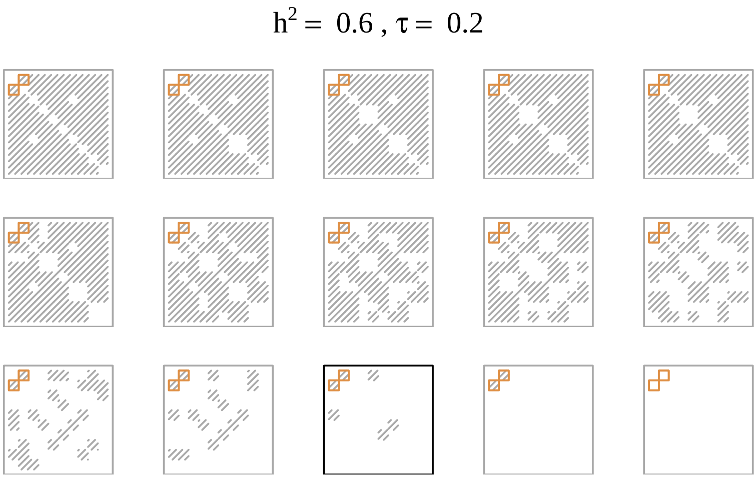

Fig. 28.

$h^2 = 0.6 , \tau = 0.3$

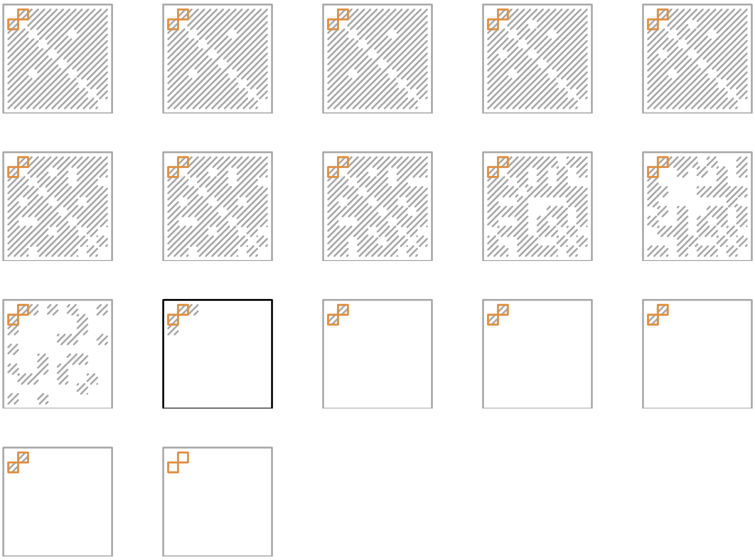

Fig. 29.

$h^2 = 0.6 , \tau = 0.4$

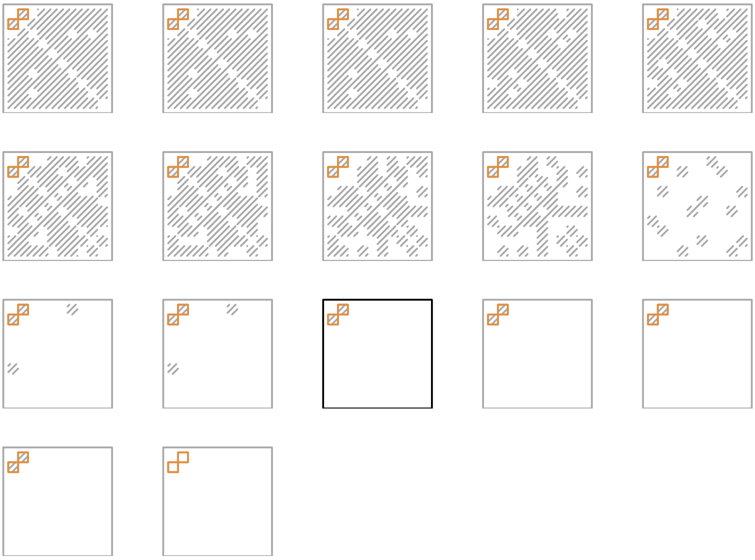

Fig. 30.

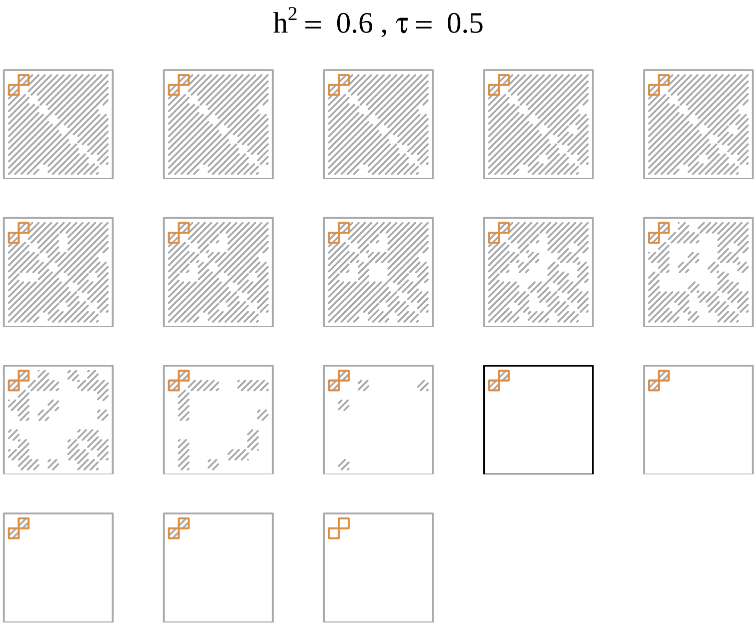

Fig. 31.

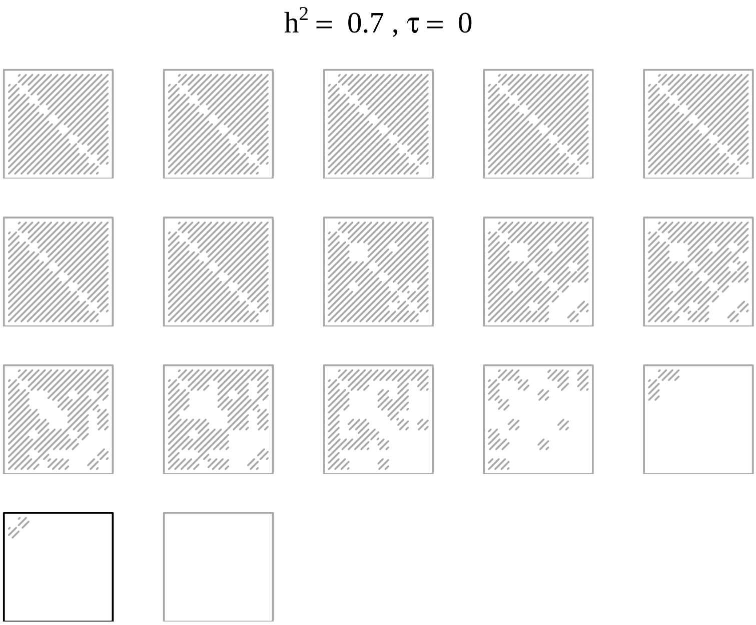

Fig. 32.

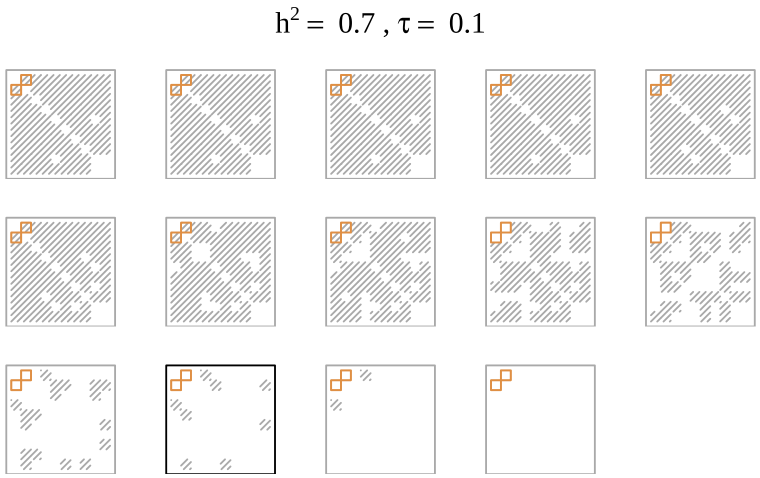

Fig. 33.

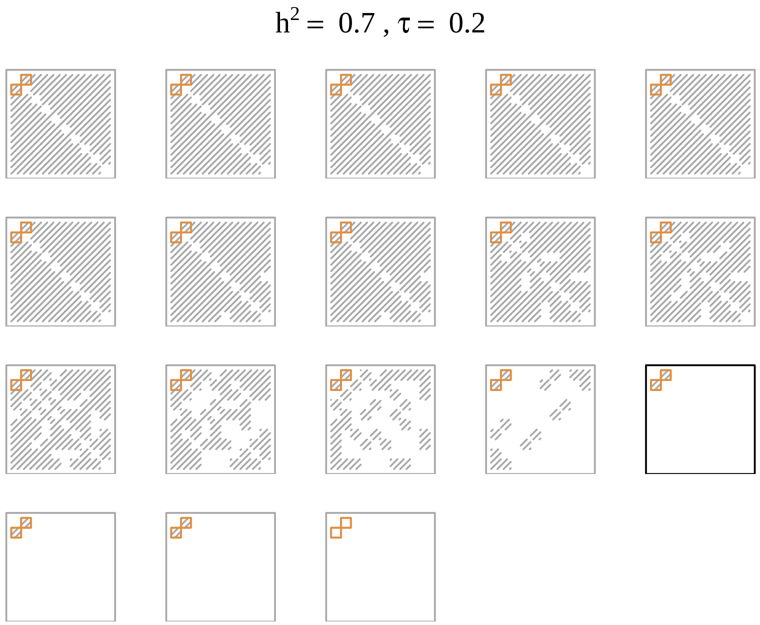

Fig. 34.

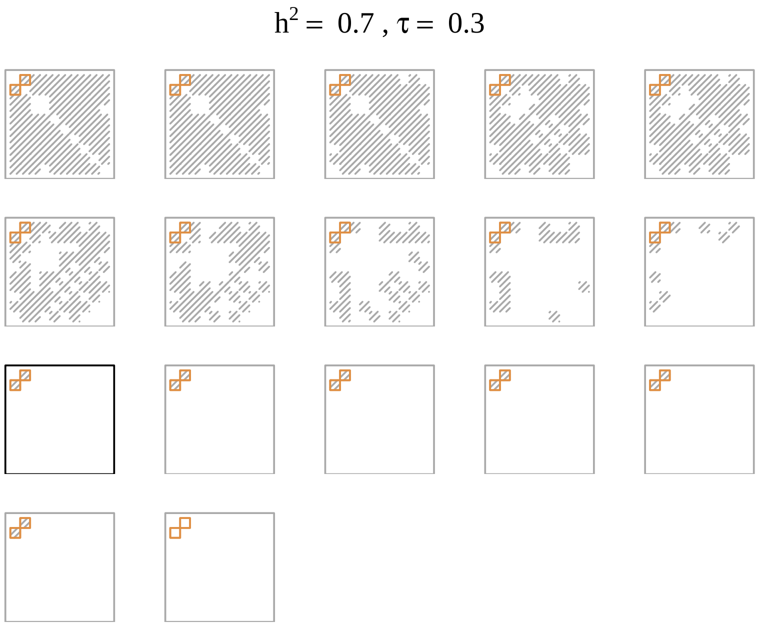

Fig. 35.

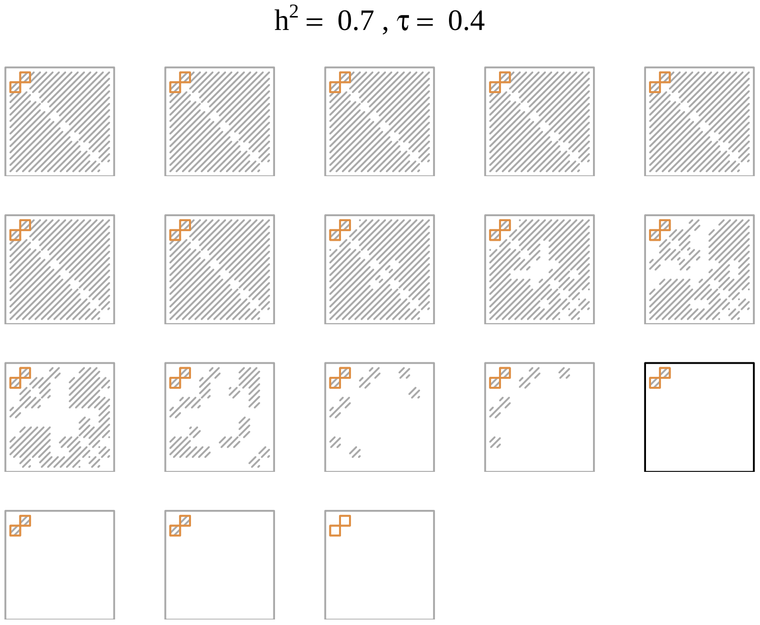

Fig. 36.

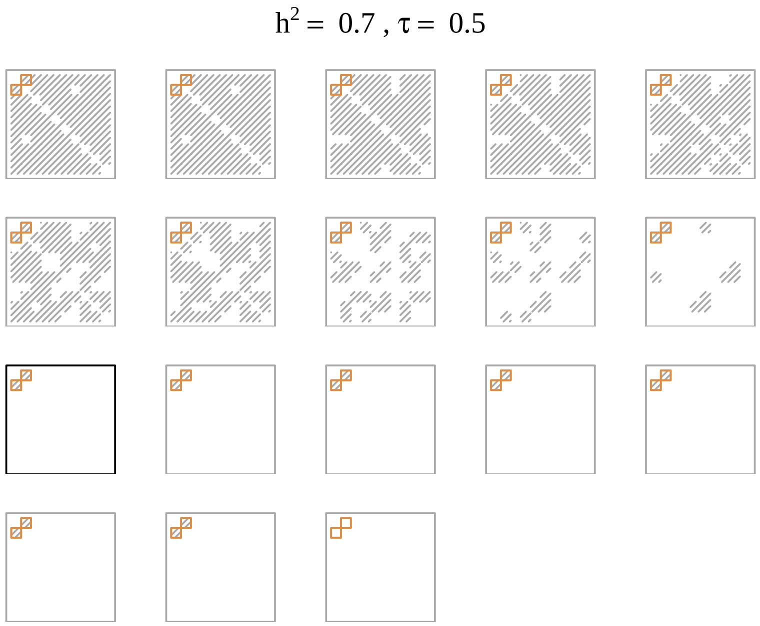

Fig. 37.

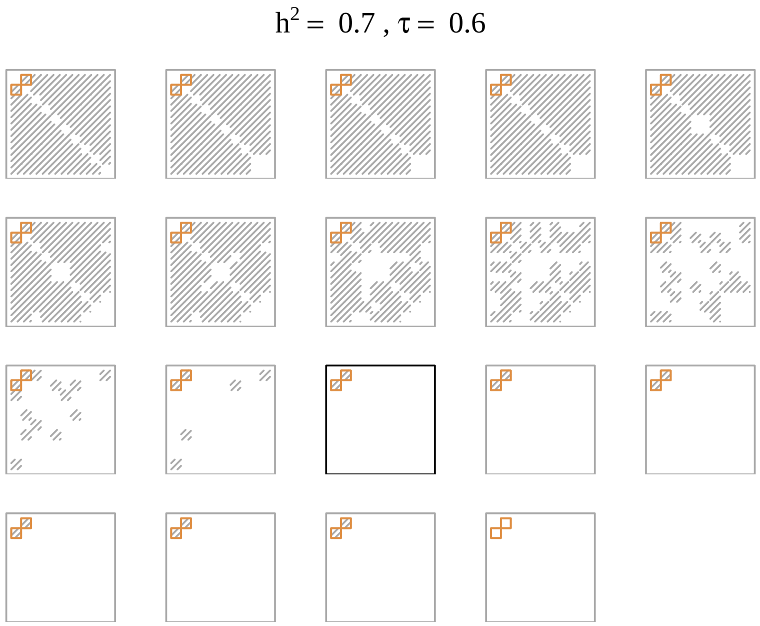

Fig. 38.

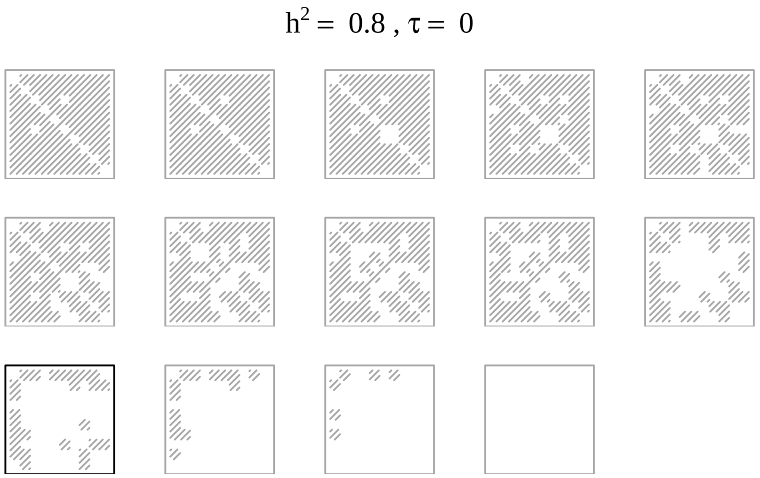

Fig. 39.

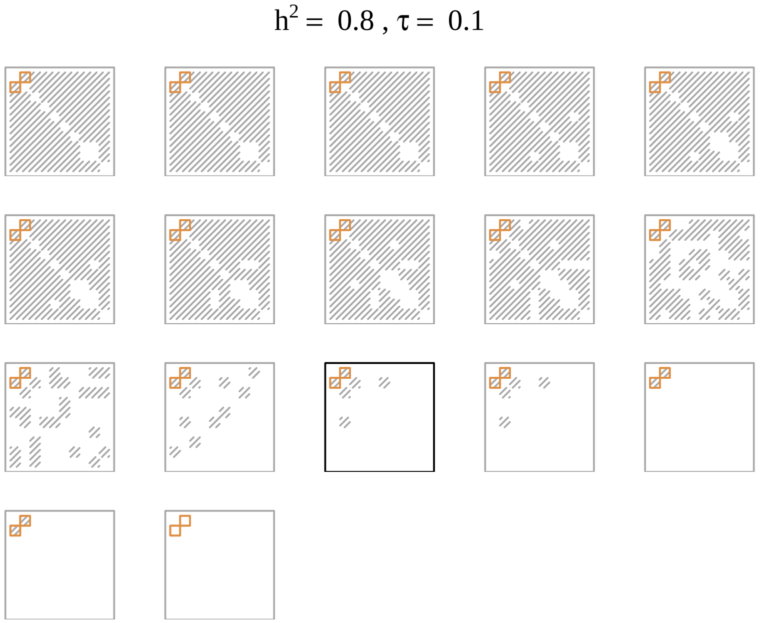

Fig. 40.

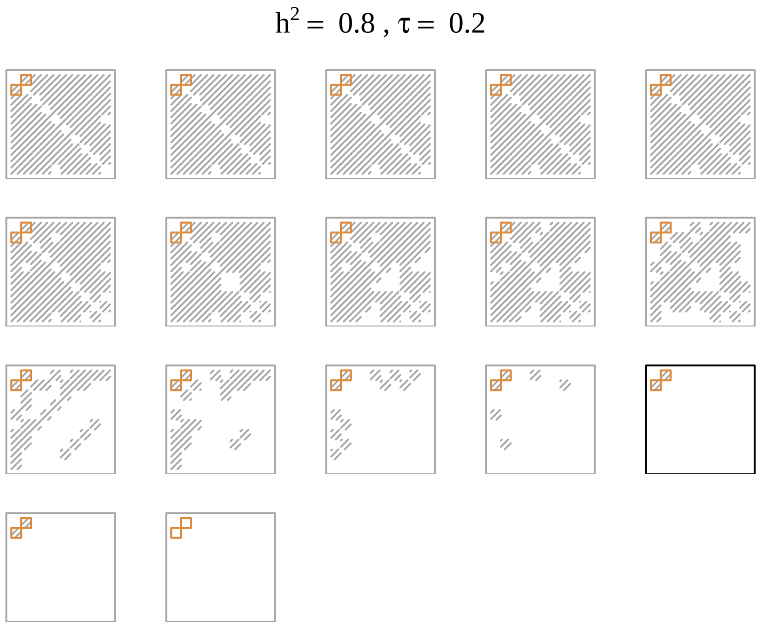

Fig. 41.

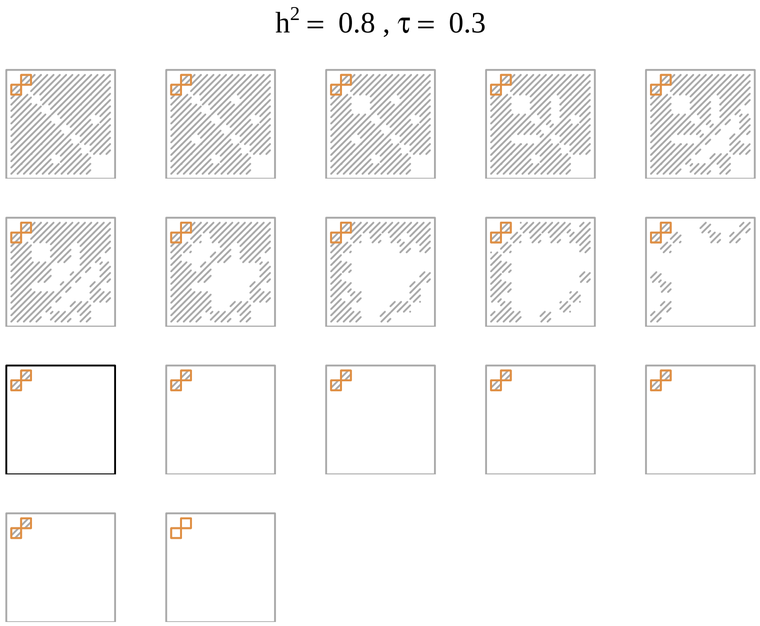

Fig. 42.

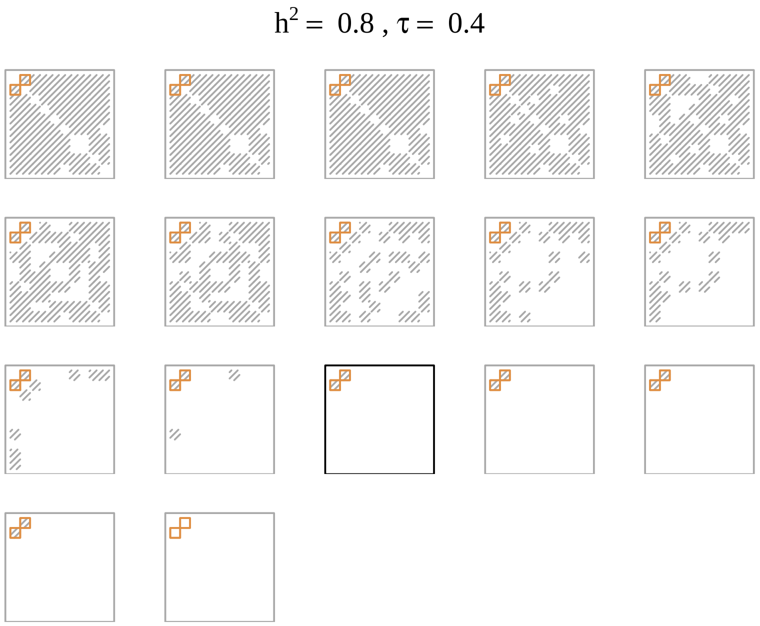

Fig. 43.

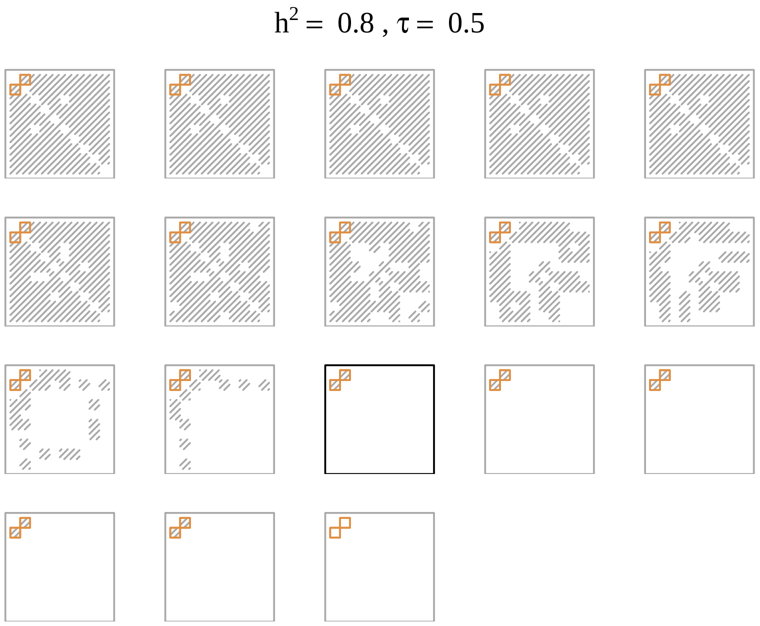

Fig. 44.

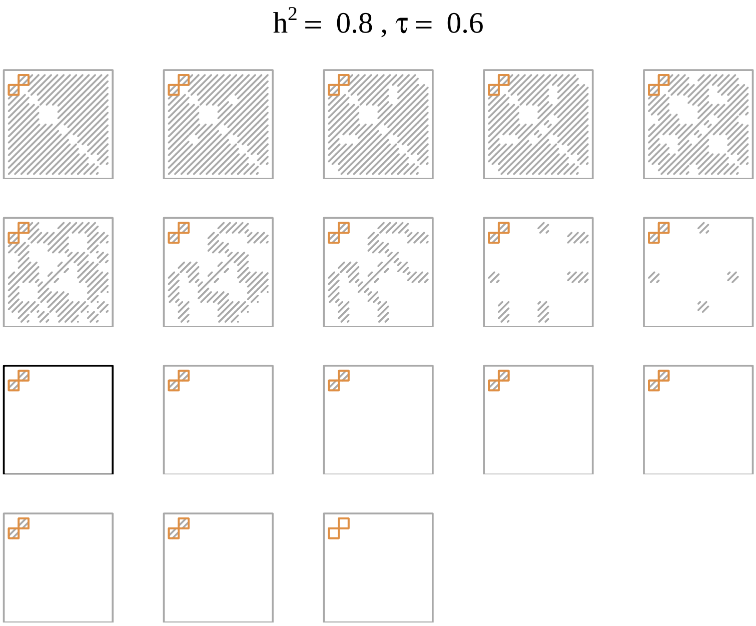

Fig. 45.

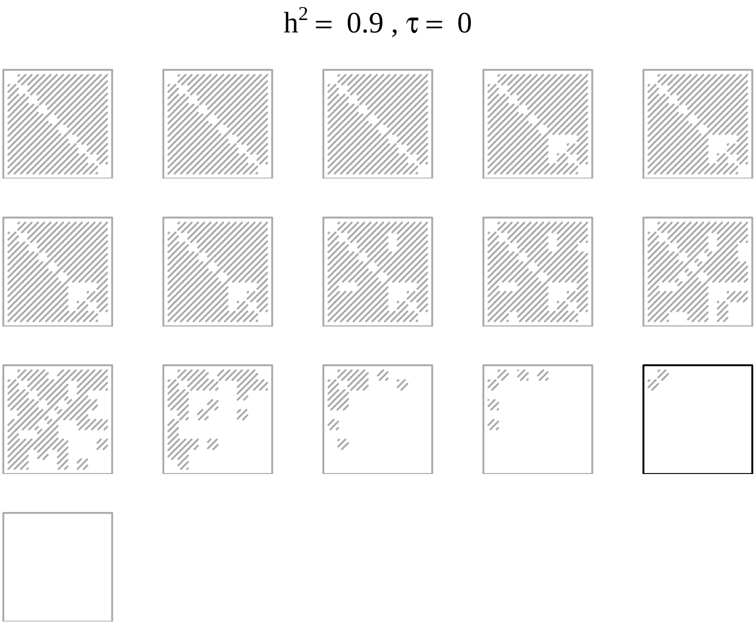

Fig. 46.

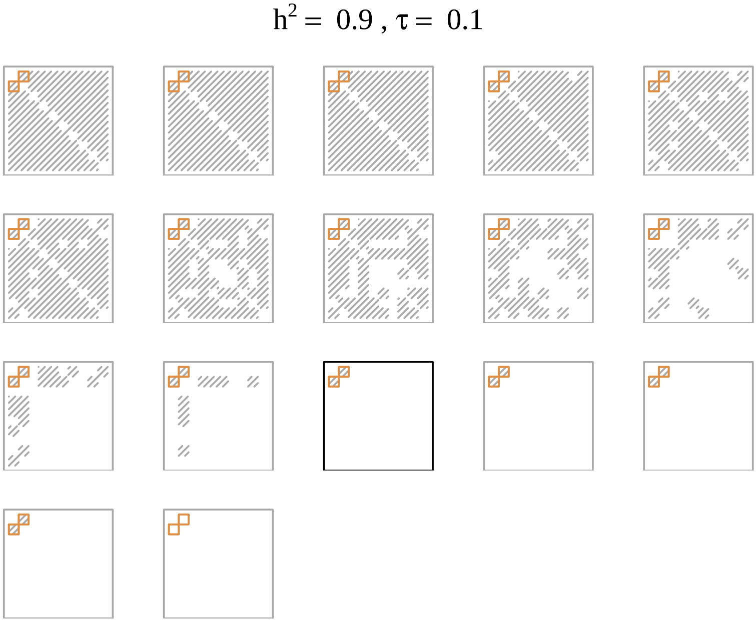

Fig. 47.

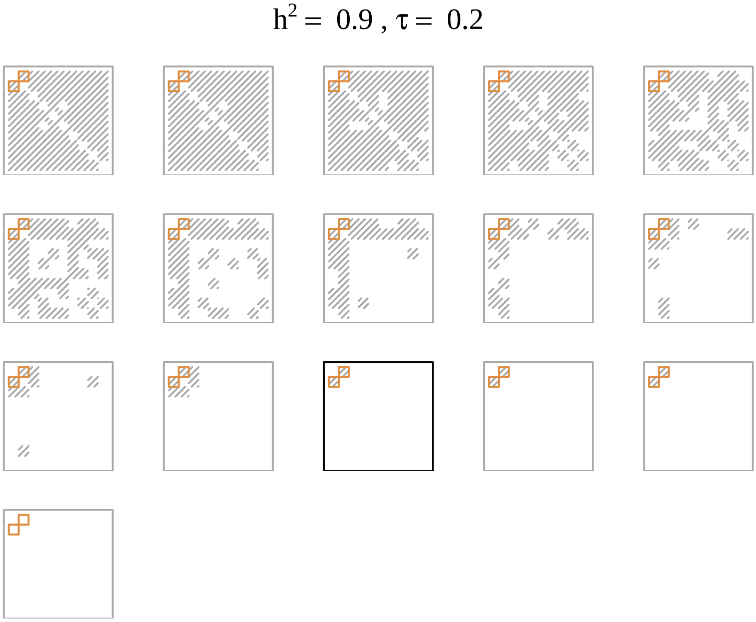

Fig. 48.

$h^2 = 0.9 , \tau = 0.3$

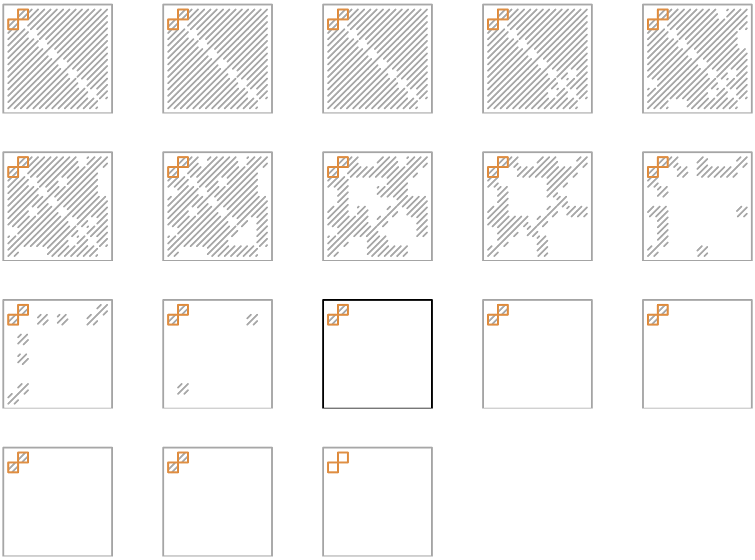

Fig. 49.

$h^2 = 0.9 , \tau = 0.4$

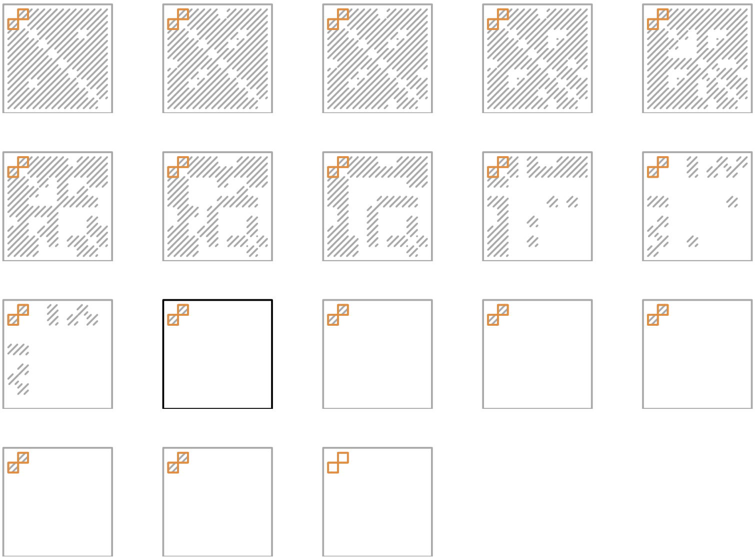

Fig. 50.

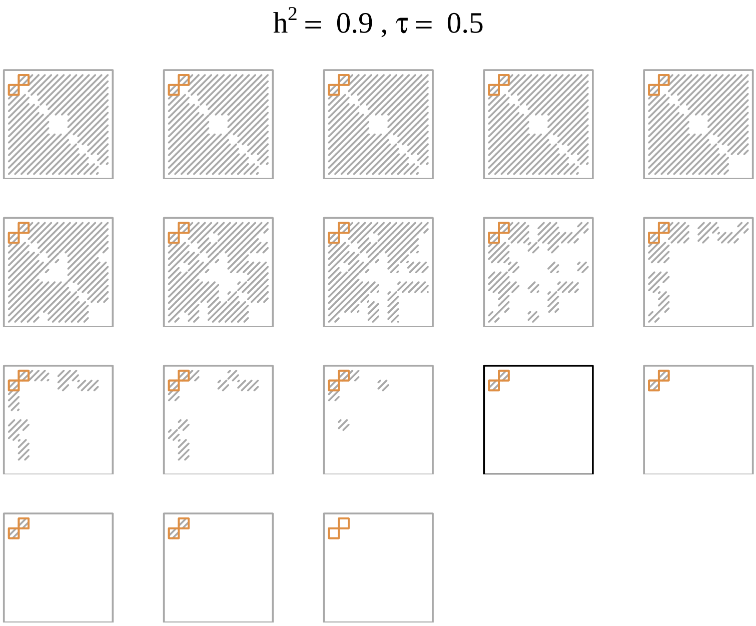

Fig. 51.

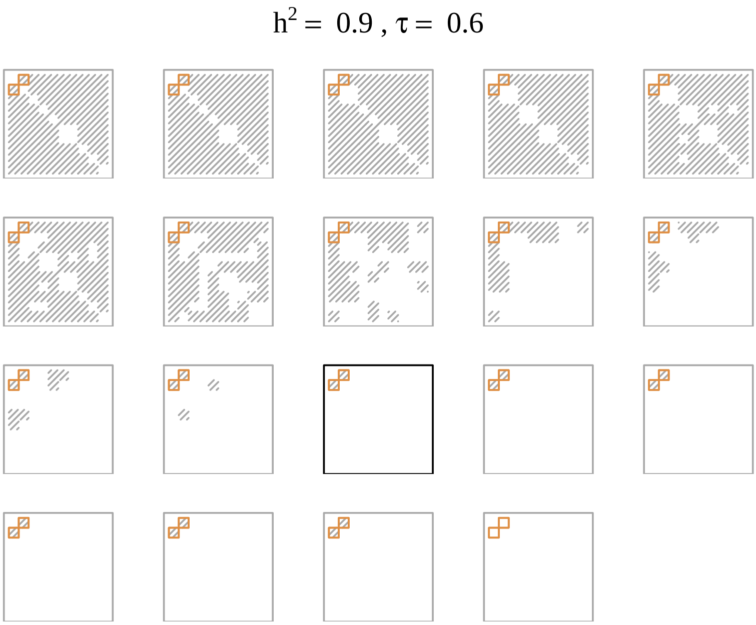

Fig. 52.

$h^2 = 0.9$, $\tau = 0.7$

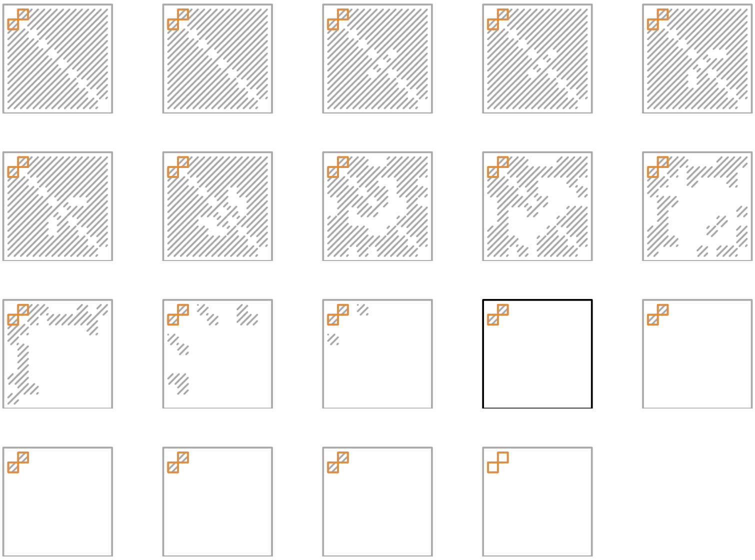

Fig. 53.
